# Hypothalamic miR-30 regulates puberty onset via repression of the puberty-suppressing factor, Mkrn3

**DOI:** 10.1371/journal.pbio.3000532

**Published:** 2019-11-07

**Authors:** Violeta Heras, Susana Sangiao-Alvarellos, Maria Manfredi-Lozano, María J. Sanchez-Tapia, Francisco Ruiz-Pino, Juan Roa, Maribel Lara-Chica, Rosario Morrugares-Carmona, Nathalie Jouy, Ana P. Abreu, Vincent Prevot, Denise Belsham, Maria J. Vazquez, Marco A. Calzado, Leonor Pinilla, Francisco Gaytan, Ana C. Latronico, Ursula B. Kaiser, Juan M. Castellano, Manuel Tena-Sempere

**Affiliations:** 1 Department of Cell Biology, Physiology and Immunology, University of Córdoba & Instituto Maimonides de Investigación Biomédica de Córdoba (IMIBIC)/Hospital Universitario Reina Sofia, Cordoba, Spain; 2 Department of Medicine, Faculty of Health Sciences, University of A Coruña, A Coruña, Spain, Instituto de Investigación Biomédica (INIBIC), University Hospital A Coruña, A Coruña, Spain; 3 Jean-Pierre Aubert Research Center, Laboratory of Development and Plasticity of the Neuroendocrine Brain, Inserm, UMR-S 1172, Lille, France; 4 Division of Endocrinology, Diabetes and Hypertension, Brigham and Women’s Hospital and Harvard Medical School, Boston, Massachusetts, United States of America; 5 Department of Physiology, Medical Sciences Building, University of Toronto, Toronto, Ontario, Canada; 6 CIBER Fisiopatología de la Obesidad y Nutrición (CIBEROBN), Instituto de Salud Carlos III, Córdoba, Spain; 7 Unidade de Endocrinologia do Desenvolvimento, Laboratório de Hormônios e Genética Molecular, LIM 42, Hospital das Clínicas, Disciplina de Endocrinologia, Faculdade de Medicina da Universidade de São Paulo, São Paulo, Brasil; 8 FiDiPro Program, Institute of Biomedicine, University of Turku, Kiinamyllynkatu, Turku, Finland; Medical Research Council, UNITED KINGDOM

## Abstract

*Mkrn3*, the maternally imprinted gene encoding the makorin RING-finger protein-3, has recently emerged as putative pubertal repressor, as evidenced by central precocity caused by *MKRN3* mutations in humans; yet, the molecular underpinnings of this key regulatory action remain largely unexplored. We report herein that the microRNA, miR-30, with three binding sites in a highly conserved region of its 3′ UTR, operates as repressor of Mkrn3 to control pubertal onset. Hypothalamic miR-30b expression increased, while Mkrn3 mRNA and protein content decreased, during rat postnatal maturation. Neonatal estrogen exposure, causing pubertal alterations, enhanced hypothalamic Mkrn3 and suppressed miR-30b expression in female rats. Functional in vitro analyses demonstrated a strong repressive action of miR-30b on Mkrn3 3′ UTR. Moreover, central infusion during the juvenile period of target site blockers, tailored to prevent miR-30 binding to Mkrn3 3′ UTR, reversed the prepubertal down-regulation of hypothalamic Mkrn3 protein and delayed female puberty. Collectively, our data unveil a novel hypothalamic miRNA pathway, involving miR-30, with a prominent role in the control of puberty via Mkrn3 repression. These findings expand our current understanding of the molecular basis of puberty and its disease states.

## Abstract 2

The microRNA miR-30 suppresses the expression of Mkrn3 (a known repressor of puberty) in the hypothalamus by binding to a highly conserved region of its 3’ untranslated region, representing a novel mechanism for finely modulating the tempo of puberty.

## Introduction

Puberty is the result of a complex series of maturational events that leads to the acquisition of reproductive capacity [1]. This intricate process is initiated at early stages of development and is controlled by multiple regulatory networks that impinge at the brain (mainly hypothalamic) centers driving the reproductive axis [2]. System biology approaches have recently suggested that the control of puberty and its timing encompasses multiple sets of genes/proteins and requires the involvement of numerous regulatory mechanisms capable of coordinating the hierarchical activation/inactivation of central stimulatory and inhibitory pathways [3–6].

MicroRNAs (miRNAs), short noncoding RNAs that repress gene expression at the posttranscriptional level, have recently emerged as relevant regulatory elements of this complex developmental process [7,8]. We have documented reciprocal changes in the hypothalamic expression of miRNAs of the let-7 family and the menarche-modulating gene, Lin28B, which suppresses the biogenesis of let-7 miRNAs [9], along the postnatal maturation preceding puberty [7]. Moreover, we also found altered Lin28B/let-7 hypothalamic ratios in rodent models of perturbed puberty induced by early developmental insults [7]. In addition, a very recent study has documented the relevant role of miRNA biosynthesis in the postnatal regulation of gonadotropin-releasing hormone (GnRH) neurons, which are indispensable drivers of puberty [8]. Likewise, increased expression of miR-200 and miR-155 in GnRH neurons during the infantile period has been shown to lift the inhibitory actions of two transcriptional repressors of GnRH expression, ZEB1 and Cebpb, to permit the progression of puberty [8]. Collectively, these findings highlight the importance of miRNAs in the modulation of genes involved in the maturational program that leads to the onset of puberty.

Very recent evidence implicates Mkrn3, a maternally imprinted gene encoding the makorin RING-finger protein-3, as an essential inhibitory component of the gene regulatory network governing puberty [10,11]. In particular, deleterious mutations of MKRN3 have been identified as the first genetic cause of central precocious puberty in boys and girls [10,11]. Consistent with these findings, a prepubertal decline in the serum levels of MKRN3 has been documented in both sexes [12,13]. In addition, two different studies have demonstrated that the hypothalamic expression of Mkrn3 mRNA and protein is significantly reduced before the onset of puberty in mice [10,14], a phenomenon that further suggests the potential relevance of the repressive actions of Mkrn3 in the central control of puberty.

While the above evidence collectively supports a relevant inhibitory role of Mkrn3 in pubertal timing, the molecular mechanisms and/or regulatory elements responsible for its precise control remain virtually unknown. Of note, it has been reported that the 3′ UTR of *MKRN3* transcript, a key element for miRNA-mediated posttranscriptional regulation of gene expression, is highly conserved in mice and humans [11,15]. However, the potential involvement of miRNAs in the control of Mkrn3 activity and their functional contribution to the timing of puberty has not been explored to date. Here, we analyzed the expression profiles of miR-30b during normal and altered puberty, and correlated its levels with those of Mkrn3. Of note, miR-30b is a member of a family of miRNAs that, according to bioinformatic tools, are putative regulators of Mkrn3. Furthermore, we evaluated the regulatory action of miR-30b on Mkrn3 activity and assessed its impact on the timing of puberty. Our results demonstrate that miR-30 is a key regulatory element of Mkrn3 expression and that the correct functioning of this novel hypothalamic miR-30/Mkrn3 pathway at early stages of development is essential for the normal initiation of puberty.

## Results

### In silico identification of binding sites for miR-30 family members at the 3′ UTR of Mkrn3

**Fig 1:**
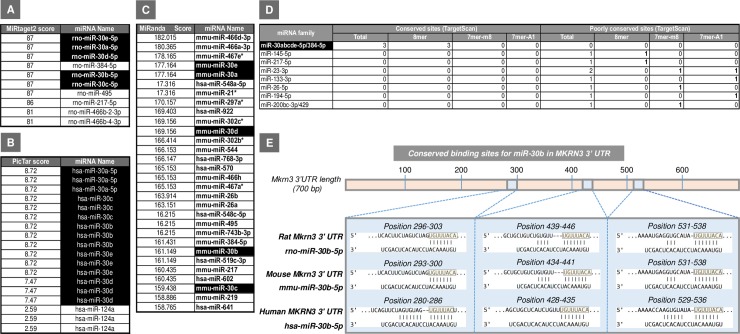
Potential miRNA regulators for *Mkrn3* identified by different bioinformatics tools: MiRtarget2 (**A**), PicTar (**B**), and MiRanda (**C**) in rat (rno), human (hsa), and mouse (mmu) are shown. In addition, the number of conserved binding sites for the potential miRNA candidates predicted by TargetScan (www.targetscan.org/) in the 3′ UTR of Mkrn3 transcript is represented **(D)**. The best miRNAs candidates are highlighted in black. The predicted binding sites for the members of the miR-30 family, including miR-30b, in rat, mouse, and human *MKRN3* 3′ UTR by using TargetScan (www.targetscan.org/) are represented in (**E**). For each species, the overall MKRN3 3′ UTR is shown (top), with detailed sequences of the predicted miR-30b target binding sites being presented below. Seed region sequences for miR-30b in the 3′ UTR of *Mkrn3* are highlighted in light orange boxes. miRNA, microRNA; *Mkrn3*, makorin RING-finger protein-3.

Using four miRNA-target prediction tools based on different bioinformatic methods (see Material and methods), we obtained several sets of potential regulatory miRNAs for the *Mkrn3* gene (**Fig 1A–1D**). Interestingly, all four of these prediction tools identified members of the miR-30 miRNA family as strong candidates for the potential regulation of *Mkrn3* gene. This prediction is based on the top "score" obtained by each of them using different miRNA-target bioinformatics algorithms, as well as the high number of predictive and conserved binding sites detected for those miRNAs in the 3′ UTR of *Mkrn3*. In particular, three seed regions for 5 members of the miR-30 family (namely, miR-30a, miR-30b, miR-30c, miR-30d, and miR-30e) are located in a highly conserved area encompassing more than 200 bp of the 3′ UTR of *Mkrn3* (**Fig 1E**).

Because of the relatively large number of miRNA candidates, including prominently all members of the miR-30 family, we decided to select one representative of this family for further studies. Among them, miR-30b was selected on the following basis: (i) its documented expression in rat hypothalamus [16]; (ii) its demonstrated regulation by sex steroids [17–19]; and (iii) the high number of predicted and conserved binding sites detected for this miRNA in the 3′ UTR of Mkrn3.

### Expression profiles of Mkrn3 and miR-30b in the hypothalamus during rat postnatal maturation

Expression analyses of Mkrn3 and miR-30b were initially applied in whole hypothalamic tissue from neonatal (postnatal day [PND] 5), mid-infantile (PND15), and peri-pubertal (PND35 in females; PND45 in males) rats. The expression of Mkrn3 mRNA progressively declined during postnatal development in the whole hypothalamus of both female and male rats. This decrease was particularly pronounced at the peri-pubertal age in both sexes. Changes of hypothalamic levels of miR-30b were the inverse of those of Mkrn3; namely, low expression was detected during the neonatal and infantile period, with a significant increase at pubertal ages in both female and male rats (**S1A and S1B Fig**).

**Fig 2:**
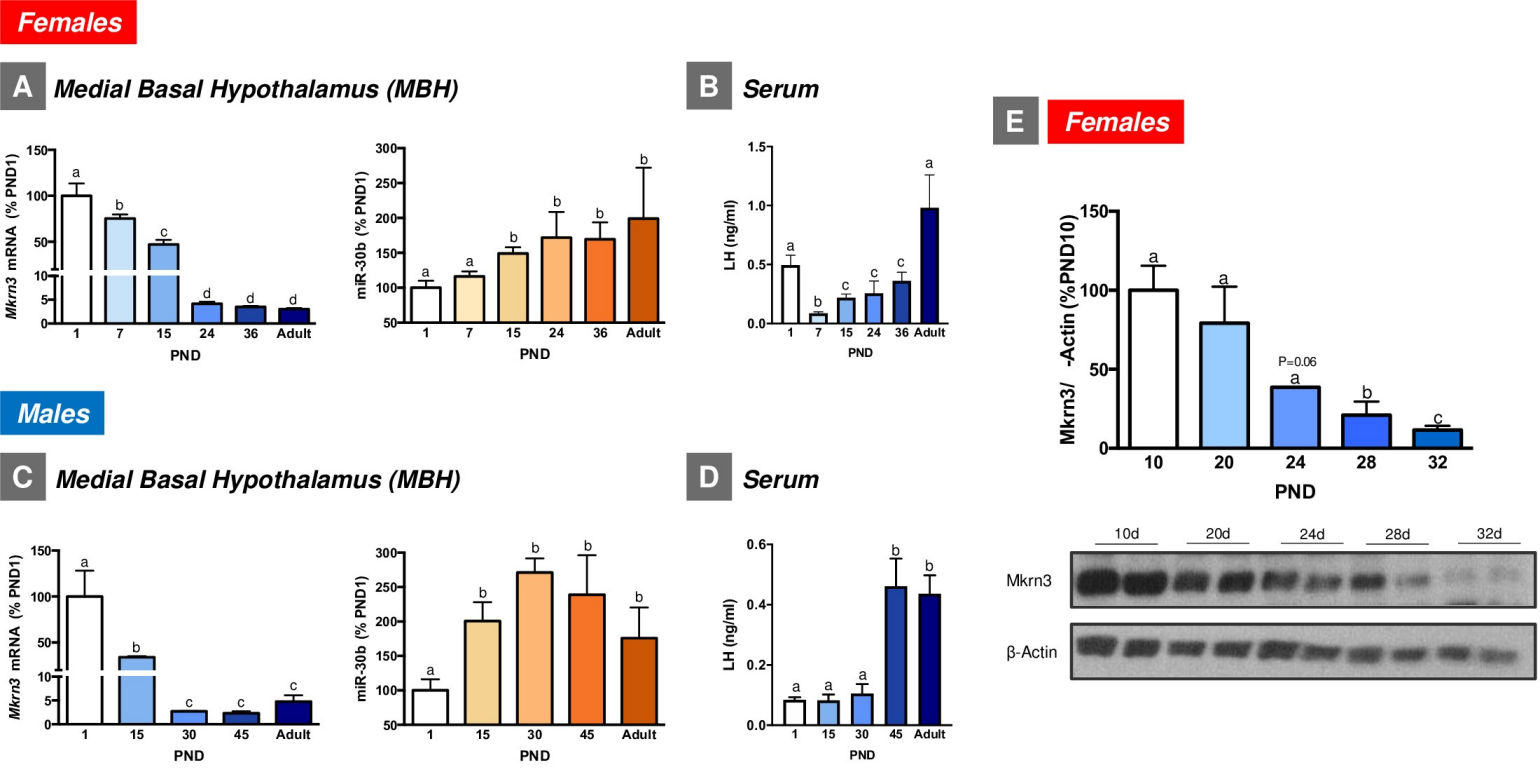
Expression profiles of Mkrn3 mRNA and miR-30b in the MBH of female (**A**) and male (**C**) rats during postnatal maturation (*n* = 5–9/group). Serum LH levels at the same stages of postnatal maturation are presented in (**B**) (females) and (**D**) (males). In addition, densitometric quantifications and a representative western blot autoradiographic image of hypothalamic Mkrn3 protein from female rats during postnatal development are shown (**E**; *n* = 5/group). Loading control (β-Actin) is also presented. Data are presented as mean ± SEM. Different superscript letters above bars indicate statistical differences; one-way ANOVA followed by post hoc Tukey test. For underlying data, see S1 Data file. LH, luteinizing hormone; MBH, medial-basal hypothalamus; Mkrn3, makorin RING-finger protein-3.

The analysis of the neuroanatomical distribution of Mkrn3 in infantile (PND10) female rats revealed intense Mkrn3-immunoreactivity in the arcuate nucleus (ARC; **S1C Fig**), a relevant hypothalamic region for the control of puberty that is contained within the medial-basal hypothalamus (MBH). Based on these data, further developmental qPCR analyses were specifically performed in the MBH of female and male rats, including additional postnatal ages. In detail, and considering the different stages of postnatal and pubertal maturation in male and female rats [20], MBH samples from female rats included PND1 (neonatal), PND7 (early-infantile), PND15 (mid-infantile), PND24 (juvenile), PND36 (peri-pubertal), and adult cyclic female rats. In turn, MBH samples from males included PND1 (neonatal), PND15 (mid-infantile), PND30 (juvenile), PND45 (peri-pubertal), and adult rats. Consistent with our initial findings, similar expression patterns of Mkrn3 mRNA were detected in the MBH of both female and male rats during postnatal development. In particular, *Mkrn3* displayed maximal expression levels during the neonatal-infantile period (PND1–15 in both male and female rats), which markedly declined during the infantile-juvenile transition (PND15–24 in female rats and PND15–30 in male rats), reached minimal levels during the peri-pubertal period (PND24–36 in female rats and PND 30–45 in male rats) and remained at low levels during adulthood (**Fig 2A** and **2C**). In contrast, miR-30b levels were the lowest neonatally and progressively increased during postnatal maturation, reaching maximum levels during the peri-pubertal period in both female and male rats (**Fig 2A** and **2C**). Consistent with Mkrn3 mRNA data, hypothalamic expression of Mkrn3 protein markedly declined during the juvenile-pubertal transition in female rats (**Fig 2E**). Interestingly, the profiles of serum levels of luteinizing hormone (LH), as surrogate marker of the activity of the key neuronal population responsible for pubertal maturation, namely GnRH neurons [21], were grossly similar to those of hypothalamic miR-30b expression, and inverse to Mkrn3 in female and male rats, with the lowest levels in the infantile period and a progressive increase towards the peri-pubertal stage, and with peak values at adulthood (**Fig 2B** and **2D**). Note that the transiently elevated LH levels detected in PND1 female rats are likely reflecting the postnatal surge of gonadotropins, reminiscent of mini-puberty in humans. For additional biochemical and phenotypic markers of normal pubertal maturation in rats, see **S2 Fig**.

### Co-expression of Mkrn3 and miR-30b in ARC Kiss1 neurons and an ARC Kiss1 cell line

Considering the reciprocal patterns of expression of Mkrn3 and miR-30b in the hypothalamus of female rats during postnatal maturation, and the prominent expression of Mkrn3 in the ARC, we searched for the cellular substrate of a potential miR-30b/Mkrn3 interplay. Given technical limitations for in situ localization of miRNAs in the rodent brain with neuronal resolution, we opted for implementing expression analyses in cells representing ARC Kiss1 neurons, as these are master elements for the central activation of GnRH neurons during postnatal/pubertal maturation [22]. First, real-time (rt)–PCR analyses were applied to the ARC-derived hypothalamic cell line, mHypoA-55, previously validated as representative of ARC Kiss1 cells [23] (**S3A Fig**). Initial analyses using specific primers and SYBR Green detection documented the expression of Mkrn3 transcript, together with Kiss1, in mHypoA-55 cells, with a threshold cycle value of 28 for Mkrn3 (**S3B Fig**). Further PCR analyses using specific TaqMan probes confirmed the expression of Mkrn3 and Kiss1 in mHypoA-55A cells and also revealed the presence of miR-30b in this ARC Kiss1 cell line, with a threshold cycle value of 26 (**S3C Fig**).

In a parallel approach, rt-PCR analyses using specific TaqMan probes were applied to Kiss1 neurons isolated from the MBH (encompassing the ARC) of a Kiss1–enhanced yellow-fluorescent protein (EYFP) reporter mouse line at the pubertal age (PND29), using well-validated protocols of fluorescence-activated cell sorting (FACS) [8] (**S4A**–**S4E Fig**). YFP-positive cells were shown to express Kiss1 and Tac2, which encodes neurokinin B (NKB), a key co-transmitter of ARC Kiss1 neurons [24,25]; yet, they did not express Npy, encoding neuropeptide Y. In contrast, no expression of Kiss1 or Tac2 was detected in YFP-negative neurons from the MBH. These features attest to the successful isolation of this Kiss1 neuronal population. ARC Kiss1 neurons did express Mkrn3, which was not detected in YFP-negative cells. Similarly, expression of miR-30b was also found in Kiss1, YFP-positive cells; yet, this miRNA was also detected in YFP-negative cells from MBH, denoting the expression of miR-30b in neurons other than Kiss1 cells at this hypothalamic site (**S4F** and **S4G Fig**). No amplification over the threshold limit was detected for any of the negative controls run in parallel for each target.

### Hypothalamic expression of Mkrn3 and miR-30b in models of perturbed puberty

**Fig 3:**
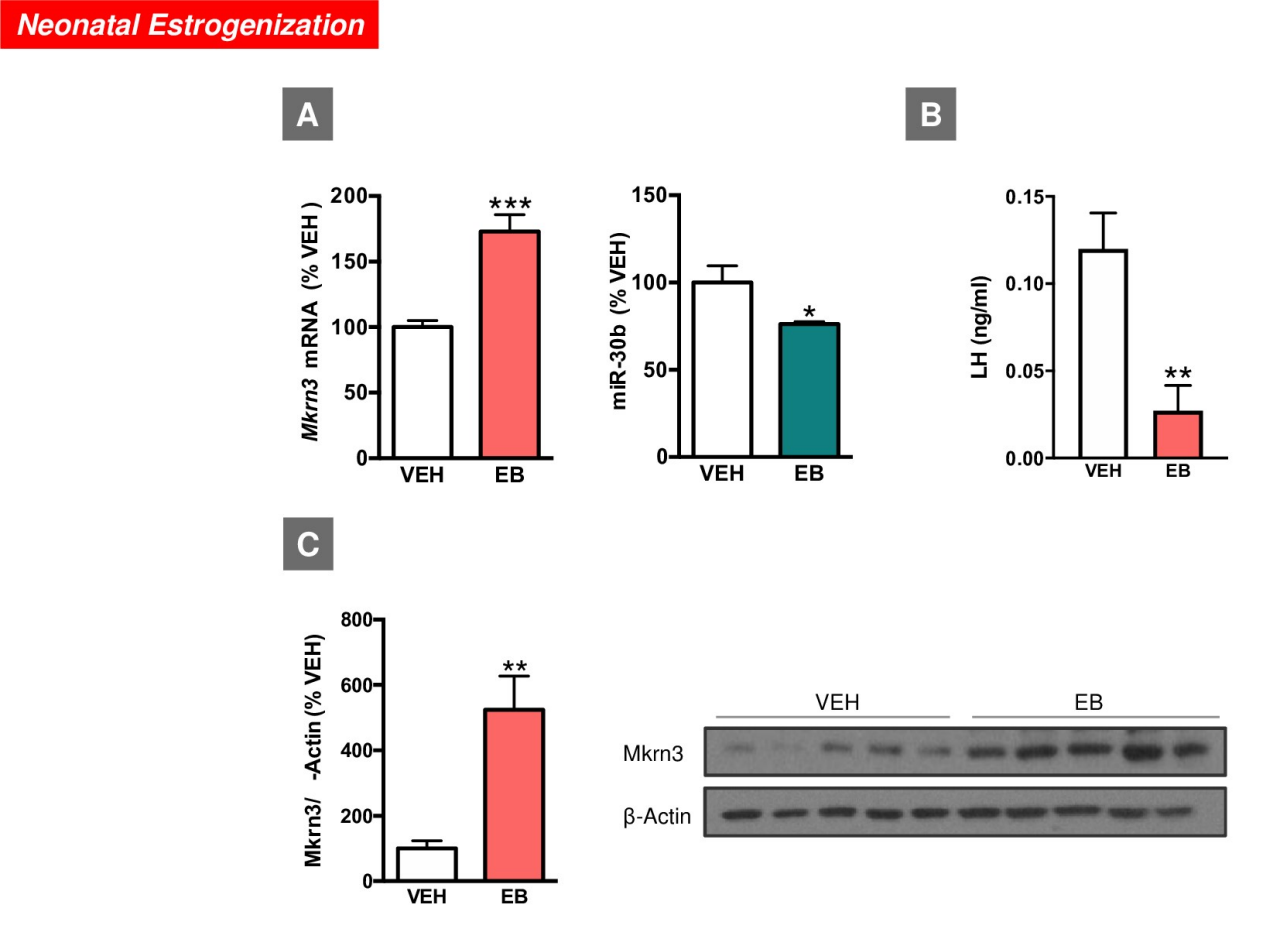
Expression profiles of *Mkrn3* mRNA and miR-30b in the hypothalamus of female rats neonatally injected (on PND1) with EB (**A**). Expression analyses were conducted at the expected time of puberty (PND35). Serum LH levels in the same experimental groups are shown in (**B**). In addition, densitometric quantification and a representative western blot autoradiographic image of hypothalamic Mkrn3 protein from female rats neonatally injected with EB are shown (**C**). Loading control (β-Actin) is also presented. Animals injected with olive oil (VEH) served as controls (*n* = 6–8/group). Data are presented as mean ± SEM. *P ≤ 0.05, **P ≤ 0.01, ***P ≤ 0.001 versus VEH; Student *t* test. For underlying data, see S1 Data file. EB, estradiol benzoate; LH, luteinizing hormone; *Mkrn3*, makorin RING-finger protein-3; PND, postnatal day; VEH, vehicle.

In agreement with previous publications [7], neonatal administration of high doses of estradiol benzoate (EB) altered pubertal maturation in female rats (see **S5 Fig**). This was evidenced by delayed or absent first estrus and decreased serum gonadotropin levels, in line with previous reports [26,27]. This phenotype was linked to changes in the hypothalamic expression of Mkrn3 mRNA and miR-30b in female rats at the expected time of puberty. Thus, on PND35, neonatally estrogenized female rats displayed significantly higher Mkrn3 mRNA and protein levels as compared with their controls (injected with vehicle: VEH). Conversely, miR-30b levels were significantly lower in EB-treated animals at the expected time of puberty compared with controls, while serum LH levels were also suppressed in EB-treated female rats at puberty (**Fig 3**).

A protocol of undernutrition during lactation, generated by breeding pups in large litters (LLs; 20 pups/litter), delayed the age of vaginal opening (VO) in female rats, in keeping with previous reports [7,28,29]. This protocol of nutritional manipulation was associated with modest alterations in the hypothalamic expression of Mkrn3 mRNA during postnatal development in early underfed female rats. Enhanced expression of Mkrn3 was detected in LL female rats at PND5 as compared with their respective controls (normal litter; NL), while no changes between NL and LL groups were observed at juvenile (PND15) and pubertal (PND35) ages. In addition, hypothalamic expression of miR-30b was not significantly altered during pubertal development in LL female rats. Nonetheless, a trend for decreased hypothalamic expression of miR-30b was observed in LL female rats at PND5, as compared with their respective NL controls (**S6 Fig**).

### Evidence for miR-30b repression of Mkrn3 from in vitro reporter analyses

**Fig 4:**
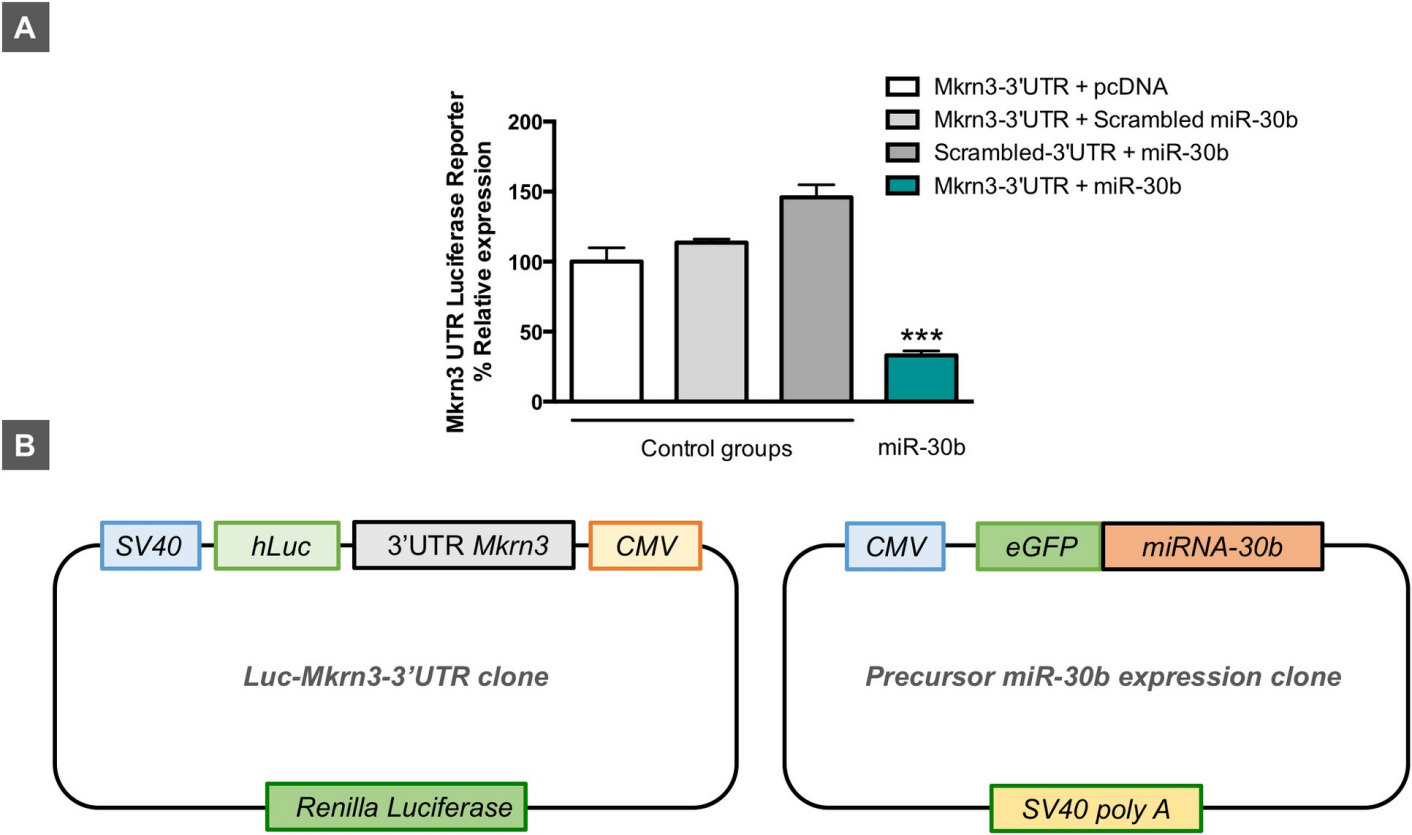
Luciferase activity of mouse *Mkrn3* 3′ UTR reporter construct after pre–miR-30b overexpression (Luc-Mkrn3-3′ UTR + pre–miR-30b) in HEK 293 cells (**A**). The following combinations of plasmids were used as controls: Luc-Mkrn3-3′ UTR + pcDNA (empty vector); Luc-Mkrn3-3′ UTR + scrambled pre-miRNA; and scrambled Luc-Mkrn3-3′ UTR + pre–miR-30b (*n* = 3 replicates/group). In addition, a schematic representation of Luc-Mkrn3-3′ UTR and pre–miR-30b clones is shown (**B**). Data are presented as mean ± SEM. ***P ≤ 0.001 versus each control group; one-way ANOVA followed by post hoc Tukey test. For underlying data, see S1 Data file. CMV, cytomegalovirus; eGFP, enhanced green fluorescent protein; Luc, luciferase; *Mkrn3*, makorin RING-finger protein-3; SV40, simian virus 40.

In order to (i) confirm in silico predictions that miR-30b targets the 3′ UTR of Mkrn3 mRNA and (ii) document the potential inhibitory role of miR-30b on Mkrn3 expression suggested by the inverse miR-30b/Mkrn3 ratios detected in animal models of normal and altered puberty, we performed Luc-Pair miR luciferase assays by cotransfecting a reporter vector harboring the 3′ UTR of mouse Mkrn3 with a precursor expression plasmid for mouse miR-30b in HEK 293 cells. As shown in **Fig 4**, cotransfection of pre–miR-30b with the reporter plasmid containing the 3′ UTR of Mkrn3 induced a marked reduction in the luciferase signal (>65%) compared with control groups transfected either with an empty vector in place of the pre–miR-30b expression plasmid, or with scrambled 3′ UTR or miRNA sequences. These data indicate that miR-30b targets the 3′ UTR of Mkrn3 and drives a repressive signal to Mkrn3 expression in vitro.

### Timed central administration of TSB-miR-30 delays puberty onset in female rats

Based on the above data, we evaluated whether miR-30 would operate as a repressor of the hypothalamic expression of Mkrn3 in vivo and, thereby, might contribute to the central control of the timing of puberty. To test this hypothesis, we used antisense-modified oligonucleotides (namely, Target Site Blocker [TSB]–miR-30) that selectively prevent miR-30 binding to its seed regions at the 3′ UTR of Mkrn3 (**S7 Fig**). The impact on pubertal timing of the central administration of a combination of three TSB-miR-30, with capacity to block miR-30 binding to each of its seed regions at the 3′ UTR of Mkrn3, was evaluated in immature female rats. Two windows of treatment were selected: (i) prepubertal, in which the TSB-miR-30 mix was injected intracerebroventricularly (icv; i.e., into the lateral ventricle) at PND24, 28, and 32, and (ii) juvenile, in which the TSB-miR-30 mix was icv injected at PND15, 22, 25, and 28 (**S7 Fig**).

Prepubertal TSB-miR-30 treatment did not modify relevant pubertal and/or metabolic parameters, including ages at VO and first estrus, ovarian and uterus weight, and body weight (BW) gain and food intake (**S8 Fig**). In good agreement, no differences in the hypothalamic levels of Mkrn3 protein were observed in TSB-miR30 animals at the time of puberty onset (PND33) (**S8 Fig**).

**Fig 5:**
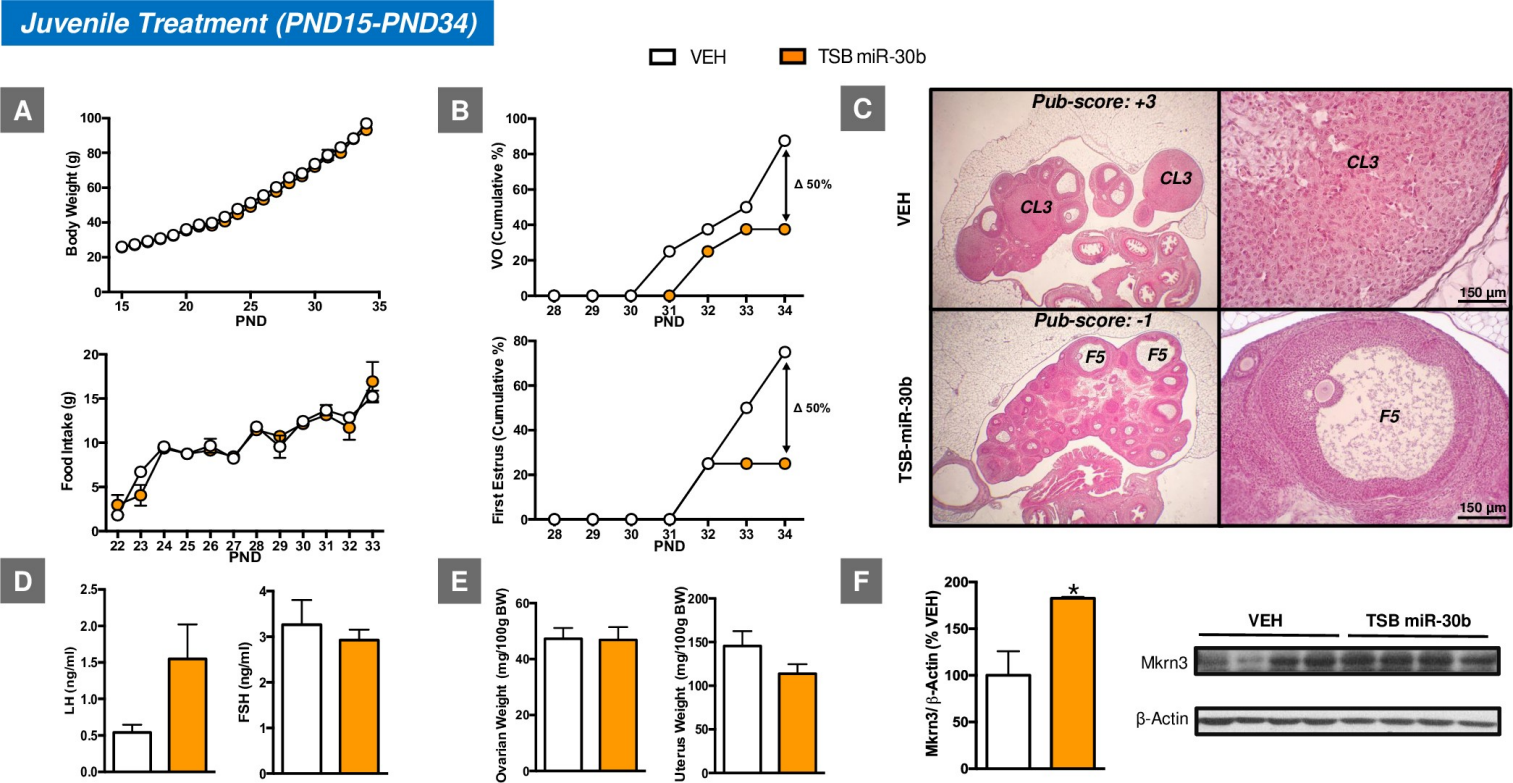
Impact of juvenile icv administration of TSB-miR-30 on the timing of puberty onset in female rats. The effects of juvenile icv treatment with TSB-miR-30 on BW and food intake (**A**), as well as relevant reproductive parameters, such as the cumulative percentage of VO and first estrus (**B**), gonadotropin levels (**D**), and ovarian weight (OW) and uterus weight (UW) (**E**) are presented (*n* = 10–12/group). In addition, representative images of ovarian maturation and its corresponding histological score of follicular development/ovulation (Pub-score) are shown (**C**). Densitometric quantifications and a representative western blot autoradiographic image of hypothalamic Mkrn3 protein from pubertal female rats subjected to juvenile icv administration of TSB-miR-30 and their controls are also presented (**F**; *n* = 4/group). Loading control (β-Actin) is also shown. Females icv injected with vehicle (VEH) served as controls. *P ≤ 0.05 versus VEH group; Student *t* test. For underlying data, see S1 Data file. BW, body weight; FSH, follicle-stimulating hormone; icv, intracerebroventricular; LH, luteinizing hormone; Mkrn3, makorin RING-finger protein-3; PND, postnatal day; TSB, Target Site Blocker; VO, vaginal opening.

In clear contrast, juvenile TSB-miR-30 treatment resulted in delayed onset of puberty, as evidenced by the decreased percentage of animals (approximately 50%) that displayed VO and first estrus by PND34 when compared with the control group (**Fig 5**). Consistent with the delayed VO and first estrus, a delay in follicular maturation and ovulatory scores was observed in the ovary. Furthermore, this pubertal phenotype was linked to increased hypothalamic levels of Mkrn3 protein at PND34, when most of the control animals showed VO and first estrus. Yet, no changes either in BW or food intake were associated with such pubertal delay. No significant changes were detected either for ovarian or uterine weights, nor were LH and follicle-stimulating hormone (FSH) levels significantly different between groups; yet, a trend for decreased uterine weight and increased LH levels was observed in TSB-miR-30 treated rats (**Fig 5**).

## Discussion

The miR-30 family has recently emerged as a pleiotropic group of regulatory miRNAs, with prominent roles in tissue and organ (including gonadal) development, as well as in disease pathogenesis, from tumorigenesis to cardiovascular and renal disorders [30]. The present data unveil a novel dimension in the physiology of this miRNA system by providing unambiguous evidence for a relevant regulatory role of miR-30 in the hypothalamic expression of the puberty-suppressing factor, Mkrn3, and its involvement in the control of the timing of puberty in rats. Considering the high penetrance of mutations of *MKRN3* as a cause of genetic forms of central precocious puberty [10,11,31,32] and the presence of three evolutionarily conserved binding sites for miR-30 family members in the 3′ UTR of *MKRN3*, our data pave the way for specific analyses of the potential pathogenic role of deregulated miR-30/MKRN3 in pubertal disorders in humans.

Mkrn3 and miR-30b displayed opposite profiles of expression in the rat hypothalamus during postnatal maturation. Notably, miR-30b was selected in our analyses as representative of the miR-30 family, as it was known to be highly expressed in the adult hypothalamus [16], and regulated by sex steroids [17–19] and nutritional cues, such as high-fat diet [16]. The observed decline in the hypothalamic expression of Mkrn3 mRNA in maturing male and female rats is fully consistent with previous studies in mice [10,14], and compatible with the concept that a reduced hypothalamic tone of Mkrn3 might be permissive for puberty to proceed. In fact, the abundant content of Mkrn3 protein in the MBH, including the ARC, in the infantile period and its drop at later developmental stages suggest that a lift in the repressive influence of Mkrn3 is required for normal progression of puberty. This is in line with the opposite profiles of circulating LH levels, as a surrogate marker of the activity GnRH neurons, as major drivers of puberty onset [21,22], which were low at the infantile period and increased at the time of puberty. The reciprocal changes in hypothalamic miR-30b expression levels, with an ascendant profile during postnatal maturation, fit well with the putative repressive role of miR-30 in the control of Mkrn3 expression, as predicted by in silico analyses and confirmed by our biochemical studies. This miR-30/Mkrn3 pathway would add to the newly identified sets of “repressors of repressors” involved in the multilayered control of puberty, as previously proposed for other key pubertal regulators, such as the Lin28/let-7 system [7], and the miR-200/Zeb1 and miR-155/Cebpb tandems [8], which control GnRH expression.

The importance of such a hypothalamic miR-30/Mkrn3 pathway in the physiological control of puberty was further attested by our in vivo studies, using timed icv administration of tailored TSB to selectively prevent binding of miR-30 to its conserved seed regions at the 3′ UTR of Mkrn3 at central levels. Thus, blocking experiments involving TSB injections during the juvenile phase (between PND15 and 28) unambiguously documented that prevention of the repressive action of miR-30 on the 3′ UTR of *Mkrn3* not only attenuates the decline of the hypothalamic content of Mkrn3 during the juvenile-pubertal transition but causes also a significant delay in the onset of puberty and partially suppresses ovarian maturation. Strikingly, this effect was developmentally sensitive, because a similar protocol of target-site blocking applied during the prepubertal transition (between PND24 and 32) failed to cause significant changes in hypothalamic Mkrn3 content or pubertal timing, a feature that further attests that the observed changes of pubertal timing after TSB-miR-30 administration specifically during the juvenile period are genuine. These findings suggest the participation of the hypothalamic miR-30/Mkrn3 pathway during discrete maturational windows in the programming of brain circuits later involved in the control of puberty onset, a possibility that is in line with results from our preclinical models of altered puberty due to early manipulations of the sex steroid milieu, in which alterations in the miR-30b/Mkrn3 ratios were detected. Of note, other forms of stress (e.g., nutritional) during early developmental windows, known to also alter the timing of puberty, failed to induce a similar pattern of persistent alteration of miR-30b/Mkrn3 ratios, suggesting a higher impact of manipulations affecting brain sexual differentiation. While our expression analyses focused on miR-30b, we cannot rule out the potential involvement of additional miRNAs in the hypothalamic control of Mkrn3 expression, as our TSB strategy prevents the binding of all miR-30 family members to the 3′ UTR. Moreover, our in silico analyses also highlighted other miRNAs with ability to bind to distinct seed regions at the *Mkrn3* gene.

Despite consistent clinical findings showing precocious puberty associated with mutations in the *MKRN3* gene, there is a conspicuous lack of knowledge on the mechanisms whereby the pubertal actions of Mkrn3 are conducted and how these are regulated. The initial assumption of a repressive role of Mkrn3 in Kiss1 neurons [10,11], which is compatible with the enrichment of Mkrn3 protein in the ARC, a hypothalamic site holding a prominent population of Kiss1 neurons [1], of infantile rats revealed by our immunohistochemical analyses, is yet to fully substantiated. In any event, our current data document that Mkrn3 and miR-30b are co-expressed in the ARC-derived Kiss1 neuronal cell line, mHypoA-55. Moreover, co-expression of Mkrn3, miR-30b, and Kiss1 was also detected in ARC Kiss1 neurons, denoted by the concurrent expression of Tac2 [25], which were isolated by FACS from the MBH of pubertal (PND29) female mice. Altogether, these data attest to the presence of miR-30 and Mkrn3 in a relevant neuronal type that produces the puberty-activating peptide, kisspeptin. Further characterization of this co-localization and its functional relevance awaits further neuroanatomical analyses, pending improvement of current methodological approaches for precisely detecting brain miRNA expression with neuronal resolution in situ. In any case, recent evidence suggests that Mkrn3 may also modulate the onset of puberty through the ubiquitination and eventual suppression of the neural differentiation factor, neural pentraxin-1 precursor (Nptx1) [14]. However, no evidence for the influence of such Mkrn3/Nptx1 pathway on elements of the gonadotropic axis has been presented yet. Regarding its regulation, very recent work has suggested that the transcription factor named downstream responsive element antagonist modulator (DREAM), predicted to bind to the 5′ UTR of Mkrn3 gene, may participate in the control of the transcriptional activity of Mkrn3 [33]. Yet, while disruption of such regulatory site may be pathogenic for central precocious puberty, direct evidence for DREAM/Mkrn3 interaction or its capacity to regulate Mkrn3 expression is yet to be presented. Interestingly, very recent evidence has preliminarily documented an interplay between miR-30 and MKRN3 in the context of gastric cancer [34], while miR-30 has been shown to modulate E3 ubiquitin ligase activity in ovarian cells [30]. Our present study adds to those previous data, unveiling the capacity miR-30 to modulate (acting at highly conserved regulatory regions of its 3′ UTR) Mkrn3 expression and demonstrating the relevance of such action on the central control of puberty. These findings expand our current understanding of the molecular basis for the regulation of puberty and may pave the way for the identification of novel causes of pubertal disorders.

## Materials and methods

### Ethics statement

All experiments and animal protocols were reviewed and approved by the Ethics Committees of the University of Cordoba and the regional government, Junta de Andalusia (ref. 31-10-14-144), and were conducted in accordance with European Union normative for the use and care of experimental animals (Directive 2013/53/UE on February 2013).

### Animals

Male and female Wistar rats bred in the vivarium of the University of Cordoba were used in this work. In addition, the Kiss1-Cre/EYFP mouse line was selectively used for analyses involving cell sorting of hypothalamic Kiss1 cells (see section “Isolation of hypothalamic Kiss1 neurons using Fluorescence-Activated Cell Sorting”). The day the animals were born was considered PND1 of age. The animals were kept under constant conditions of light (14 hours of light, from 7 AM) and temperature (22°C), unless otherwise stated. The animals were weaned on PND21 and were provided with free access to tap water and pelleted food (A04, Panlab), unless otherwise indicated. As general rule, the animals were randomly assigned to the different experimental groups before initiation of procedures.

### Experimental design

#### Hypothalamic expression of Mkrn3 and miR-30b during normal and altered puberty

The first set of experiments was focused on the analysis of the hypothalamic expression of Mkrn3 and miR-30b, for which our in silico analyses revealed the presence of three seed regions in a highly conserved area of the 3′ UTR of *Mkrn3*, during normal pubertal development and in two different models of altered puberty induced by early developmental insults: neonatal estrogenization and early postnatal underfeeding. In addition, the neuroanatomical distribution of Mkrn3 was assessed at the infantile period, and co-expression of Mkrn3 and miR-30b was evaluated in Kiss1 cells.

In Exp. 1, the hypothalamic expression of Mkrn3 and miR-30b was studied during postnatal maturation. The expression profiles of Mkrn3 mRNA and miR-30b were determined in the hypothalamus of female and male rats at different age points during postnatal maturation [20]: neonatal (PND1), early-infantile (PND7), mid-infantile (PND15), juvenile (PND24 in females; PND30 in males), peri-pubertal (PND36 in females; PND45 in males), and adult (PND75) ages (*n* = 5–9/group). In addition, Mkrn3 protein content in the hypothalamus of female rats was analyzed at equivalent age points of postnatal maturation, which included the infantile (PND10), juvenile (PND20 and PND24), prepubertal (PND28), and peri-pubertal (PND32) stages (*n* = 5/group) [20]. Note that minor differences in the specific age points used for RNA and protein analyses are due to the different availability and methodological processing of tissue samples from different groups of animals for qPCR and western blot (WB). Yet, they cover similar periods of postnatal maturation in male and female rats. The hypothalamus and/or MBH were collected after euthanasia, frozen in liquid nitrogen, and stored at −80°C for subsequent qPCR or WB analyses. In addition, (i) external markers of puberty onset, such as the age of VO and first estrus in females and the age of preputial separation (PS) in males, were daily monitored during postnatal development; (ii) reproductive organs’ weights (ovary, uterus, testis, and prostate) were recorded at the age of first estrus and PS; and (iii) blood samples were collected for hormonal determinations (LH and FSH) after euthanasia of the animals at different stages of postnatal maturation, including the day of VO and first estrus in females, and the day of PS in males.

In Exp. 2, hypothalamic expression of Mkrn3 and miR-30b was studied in models of altered puberty. In order to provide further evidence for the putative roles of this system in the maturational program leading to puberty, we conducted a comprehensive series of expression analyses in the following preclinical models of perturbed puberty: (i) Neonatal estrogenization model: Neonatal female rats were exposed to high doses of EB as a model of disrupted brain sexual differentiation and altered puberty. Alterations of the sex steroid milieu during the critical neonatal period of sexual differentiation are known to disrupt pubertal maturation and gonadotropic function later in life [35]. Female rats (*n* = 6/group) were injected subcutaneously (sc) on PND1 with olive oil alone (100 μL) (VEH: control group) or EB dissolved in olive oil (EB; 100 μg/rat). Hypothalami were collected after euthanasia, frozen on liquid nitrogen, and stored at −80°C for further qPCR and/or WB analyses. In addition, the occurrence of VO and first estrus were daily monitored, and blood samples were collected for LH determinations after euthanasia of the animals at the expected time of puberty (PND35). (ii) Early postnatal underfeeding model: Female rats bred in LLs (20 pups/dam) were used as a model of delayed puberty [28]. This model has been proposed to mimic nutritional challenges during the last trimester of human gestation [36]. After weaning, the rats were reared with ad libitum access to water and food. Subsets of rats were sacrificed at PND5, 15, and 35. Hypothalami were collected after euthanasia, frozen on liquid nitrogen and stored at −80°C for further qPCR analyses.

In Exp. 3, the distribution of Mkrn3 was studied in the hypothalamus of infantile female rats. To this end, IHC assays were performed in female rats at PND10. This age was selected on the basis of the high expression levels obtained for Mkrn3 mRNA in Exp. 1.

In Exp. 4, we analyzed the co-expression of *Mkrn3* and miR-30b in ARC Kiss1 neurons. In order to provide qualitative evidence for the potential co-expression of Mkrn3 and miR-30b in Kiss1 neurons, a relevant neuronal cell type of the ARC, with essential roles in the control of puberty [22], two approaches were used. First, qPCR analyses were applied to an immortalized Kiss1 cell line, the mHypoA-55, derived from and representing the ARC population of Kiss1 neurons [23]. Second, we isolated Kiss1 neurons from the ARC of a reporter mouse line, expressing EYFP selectively in Kiss1 cells, using FACS, according to well-validated protocols [8]. Peri-pubertal (PND29) Kiss1-YFP mice were used for FACS and subsequent qPCR analyses, as described below.

#### Analysis of the functional role of miR-30b/Mkrn3 pathway in the central control of puberty

The next set of experiments aimed to provide functional evidence for the potential role of the miR-30/Mkrn3 pathway in the central control of puberty by using both in vitro and in vivo experimental approaches.

In Exp. 5, the effect of miR-30b overexpression on 3′ UTR Mkrn3 reporter was analyzed in HEK 293 cells. To this end, a Luc-Pair miR luciferase assay was used to document the putative targeting of the mouse *Mkrn3* 3′ UTR by miR-30b. *Mkrn3* 3′ UTR luciferase reporter construct (217MmiT030494-MT06), precursor expression plasmid for mmu-miR-30b (217MmiR3458-MR04), control 3′ UTR expression vector (217CmiT000001-MT06) and precursor miRNA scrambled control plasmid (217CmiR0001-MR04) were all obtained from Genecopoeia (Rockville, MD). An empty expression vector, pcDNA3.1, was also used in the assays. Renilla luciferase was included on the 3′ UTR Mkrn3 construct and was used to normalize transfection efficiency.

HEK 293 cells were used for the luciferase reporter experiment. The cells were maintained in DMEM (high glucose, Gibco, Life Technologies, Grand Island, NY) and supplemented with 10% fetal bovine serum (FBS), 2 mM L-glutamine, and 1% (v/v) penicillin/streptomycin at 37°C in a humidified atmosphere containing 5% CO2. For the assays, the cells were seeded onto 24-well plates (1 × 10^5^ cells/well) in DMEM containing 10% FBS. After 24 hours, cells were transiently cotransfected with (i) *Mkrn3* 3′ UTR-luc reporter and pcDNA3.1, (ii) *Mkrn3* 3′ UTR-luc reporter and scrambled miRNA vector, (iii) 3′ UTR control vector and mmu-miR-30b expression vector, and (iv) *Mkrn3* 3′ UTR-luc reporter and mmu-miR-30b expression vector at a concentration ratio 1:4 (250 ng:1,000 ng), respectively. Transfections were performed using three replicates for each experimental condition. Polyethylenimine (PEI, #23966; Polysciences, Warrington, PA) was used as a transfection reagent at a concentration ratio of 1 μg DNA:3 μL PEI. After 5 hours of transfection, the medium containing transfection reagents was replaced by fresh DMEM; 24 hours later, the Renilla and Firefly luciferase activities were quantified by the Luc-Pair miR luciferase assay kit (217LPFR-M010, Genecopoeia) and measured on an automatic tube luminometer AutoLumat LB9510 (Berthold Technologies GmbH, Germany). Expression of the 3′ UTR reporter was measured by Firefly luciferase luminescence and normalized using the control Renilla-luc luminescence.

In Exp. 6, the impact on puberty onset of the blockade of miR-30 actions on 3′ UTR of Mkrn3 was studied in vivo. In order to provide in vivo evidence for the functional role of an miR-30/Mkrn3 pathway in the central control of puberty, we selectively prevented the binding of miR-30 to the 3′ UTR of the *Mkrn3* gene at central levels during the juvenile-pubertal transition. To this end, we icv injected custom-designed miRNA TSBs (miRCURY LNA microRNA Power Target Site Blocker in vivo use; Exiqon) in immature female rats. These miRNA TSBs are antisense-modified oligonucleotides that bind to the miRNA target site(s) of a given mRNA and prevent miRNAs from gaining access to that site. Of note, these miRNA TSBs do not catalyze RNase H-dependent degradation of the mRNA. Therefore, miRNA TSBs administration would avoid the (mostly repressive) actions of the miRNA, thereby inducing increased expression of the protein encoded by the targeted mRNA. MiRNA TSB are designed to cover a larger sequence than the miRNA seed region to ensure target specificity. Three sequences were generated to protect the three miR-30 binding sites in the Mkrn3 3′ UTR (TSB-miR-30). They were mixed at equimolar ratios (300 pmol/5 uL) and icv injected during two windows of postnatal development: (i) prepubertal treatment, in which TSB-miR-30 was icv injected at PND 24, 28, and 32; and (ii) juvenile treatment, in which TSB-miR-30 was icv injected at PND15, 22, 25, and 28. For specific details concerning icv administration, see the section “Cannulation and icv administration” below. BW, food intake, VO, and first estrus were daily monitored in both treatments. In addition, ovarian and uterus weights were recorded, and blood, hypothalamic, and ovarian samples were collected after euthanasia (PND33 for prepubertal treatment and PND34 for juvenile treatment). Mkrn3 protein levels were assayed by WB in hypothalamic samples as proxy marker of the putative blocking action of TSB-miR-30 at the 3′ UTR of the Mkrn3 gene.

#### General experimental and analytical procedures

Regarding sample collection, for analyses involving rt PCR and WB, hypothalami were dissected out immediately upon the decapitation of the animals by a horizontal cut approximately 2 mm in depth, with the following limits: 1 mm anteriorly from the optic chiasm, the posterior border of mammillary bodies, and the hypothalamic fissures. In addition, selected expression analyses were conducted using the MBH. Dissection of this hypothalamic region was conducted as previously recommended [37]: the MBH was dissected by two lateral cuts along the hypothalamic sulci, one posterior cut along the rostral border of the mammillary bodies, and one anterior cut immediately behind the optic chiasm. The tissues were frozen in liquid nitrogen and stored at −80°C until used for PCR or WB analyses. For hormone assays, blood samples were obtained by decapitation of the animals. Serum samples were separated by centrifugation at 3,000*g* for 20 minutes and stored at −20°C until they were used for hormone determinations.

Regarding phenotypic evaluation of pubertal maturation, in addition to phenotypic analyses in male and female rats during normal postnatal/pubertal maturation (see section “Hypothalamic expression of Mkrn3 and miR-30b during normal and altered puberty” above), somatic and reproductive indices of female pubertal development were evaluated in TSB experiments as previously described [29], including (i) BW; (ii) age of VO, a consensus external marker of puberty in rodents; (iii) age of first estrus, a marker of first ovulation in rodents; (iv) uterine and ovarian weight; and (v) serum LH and FSH levels. VO was monitored daily from the beginning to the end of the experiments. At that time, uterine and ovarian weight were recorded and serum hormone levels were assayed. Once VO occurred, vaginal lavages were performed daily to identify the occurrence of the first estrus, which in rodents is manifested by a predominance of cornified cells.

Regarding ovarian histology, ovarian samples (including the oviduct and the tip of the uterine horn) were fixed for at least 24 hours in Bouin solution and subjected thereafter to dehydration and embedded in paraffin wax. Serial (7-μm-thick) sections were cut, stained with hematoxylin and eosin, and evaluated under the microscope using previously validated procedures in our group [38]. Precise assessment of pubertal progression was conducted using a scoring method (Pub-Score) recently validated by our group [39]. According to this method, precise dating of pubertal maturation is based on the combined analysis of follicular development and corpus luteum dynamics (the latter, for animals that have completed first ovulation). To this end, in non-ovulating animals, the most advanced healthy antral follicle class, from small follicles measuring less than 275 μm in diameter to antral follicles (from F1 to F5), was determined, allowing dating of prepubertal maturation from stages −5 to −1. In addition, for animals that had undergone ovulation, dating of corpora lutea (CL) as a morphological sign of ovulation was also implemented, based on major histological features, allowing staging of pubertal timing at one-day intervals, from +1 (equivalent to CL1) to +5 (equivalent to CL5). Note that negative scores denote expected days until first ovulation, whereas positive scores indicate days after the first ovulation.

Regarding cannulation and icv administration, for intracerebral injections, standard procedures of cannulation of the lateral cerebral ventricle, followed by icv administration of different compounds, were implemented in immature female rats [40]. Animals were cannulated 24 hours before the beginning of the pharmacological studies unless otherwise stated. To this end, cannulas (INTRAMEDIC polyethylene Tubing, Becton Dickinson, Sparks, MD) were inserted to a depth of 2 mm beneath the surface of the skull, with an insert point at 1 mm posterior and 1.2 mm lateral to Bregma according to a rat brain atlas [41]. After cannulation, the animals were housed in individual cages until the end of the experiments. Daily inspection of the cannulae was conducted in each animal in order to exclude those showing obvious displacement or de-attachment. Note that when the treatments were started at the mid-infantile period, the compounds were directly administered into the lateral cerebral ventricle specifically on PND15, after opening a circular hole in the skull of the diameter of the cannula at the above coordinates; injection on PND15 was applied free-handed, without cannula implantation. Thereafter, cannulae were implanted after weaning, at PND21, and successive injections were applied via the cannula as described above.

Regarding cell culture and reagents, the hypothalamic-derived cell line mHypoA-55 was used to model ARC Kiss1 neurons. This cell line was originated from hypothalamic tissue microdissection isolating the ARC of 2-month-old C57BL/6 female mice. Tissue homogenates were cultured and hypothalamic primary cultures were immortalized by retroviral transfer of the SV40 T-Ag and subcloned through successive dilutions of trypsinized cells into 96-well tissue culture plates coated with poly-l-lysine until the optimal dilution with only 1 or 2 cells/well. Finally, immortalized Kiss1-expressing cell lines were isolated in order to generate clonal cell lines, which were subjected to molecular phenotyping [23]. For expression analyses, mHypoA-55 cells were grown in a monolayer in DMEM supplemented with glucose (1 mg/mL), 5% FBS (Sigma-Aldrich), and 1% penicillin/streptomycin (Gibco, Burlington, Ontario, Canada). Cells were maintained at 37°C and 5% CO_2_, as previously described [23], until used for RNA isolation and qPCR analyses.

Regarding the isolation of hypothalamic Kiss1 neurons using FACS, a mouse line selectively expressing YFP in Kiss1 cells was generated by crossing the well-validated mouse line carrying Cre recombinase expression under the Kiss1 promoter, the Kiss1-Cre^+/−^ mouse [42], with a mouse line named R26R-EYFP that harbors a loxP-flanked STOP sequence followed by the EYFP gene inserted into the Gt(ROSA)Sor locus (B6.129X1-*Gt(ROSA)26Sor*^*tm1(EYFP)Cos*^*/J*; stock# 006148; The Jackson Laboratory; Bar Harbor, ME), thus allowing Cre-mediated STOP sequence deletion and EYFP expression in Kiss1 neurons of the double mutant offspring.

MBH tissue encompassing the ARC of the Kiss1-Cre/YFP mouse at the pubertal stage (PND29) was microdissected and enzymatically dissociated using a Papain Dissociation System (Worthington, Lakewood, NJ) to obtain single-cell suspensions. FACS was applied to these suspensions using a FACS Aria III Sorter (BD Biosciences). The FACSDiva software (BD Biosciences) was used for the sort, and Kaluza software (Beckman Coulter) was used for the analysis. The sort decision was based on measurements of YFP fluorescence (excitation: 488 nm; detection: YFP band-pass 530/30 nm). First, cells were gated for forward scatter (FSC-A) and side scatter (SSC-A) to exclude cellular debris, and then aggregates were removed by selecting singlets using an FSC-A versus FSC-H plot (**S4 Fig**). Finally, a YFP-A versus 780/60-nm filter plot was used to separate YFP-positive and -negative cells. The YFP-negative cells from cortex were used as a negative control to set gates properly (**S4 Fig**). YFP-positive and -negative cells were sorted directly into 10 μL extraction buffer (0.1% Triton X-100 [Sigma-Aldrich] and 0.4 U/μL RNaseOUT [Life Technologies]).

Regarding reverse transcription (RT) and rt PCR (mRNA and miRNA) assays, RT-PCR was used to assess mRNA and miRNA expression in hypothalamic tissue and Kiss1 cells, as described below.

For analyses of mRNA expression in hypothalamic rat tissues, total RNA was extracted from the whole hypothalamus or the MBH using TRIsure isolation reagent (Bioline Reagents, UK) according to the manufacturer’s instructions. The RNA concentration of each sample was determined using a Nanodrop ND-1000 v3.5.2 spectrophotometer (Nanodrop Technology, Cambridge, UK).

For SYBR Green RT-qPCR analyses, 1 μg total RNA was DNAse-treated following the manufacturer’s recommendations (172–5034; iScriptTM gDNA Clear cDNA Synthesis Kit; Bio-Rad Laboratories), to remove potential genomic DNA contamination. Each DNA digestion consisted of 1 μg total RNA and 2 μL DNAse master mix (iScript DNAse + DNAse Buffer) in a final volume of 16 μL. This reaction mix was incubated at 25°C for 5 minutes (DNA digestion) and 75°C for 5 minutes (DNAse inactivation). Then, each DNAse-treated RNA sample was reverse transcribed in a reaction consisting of 16 μL DNAse-treated RNA template and 4 μL iScript Reverse Transcription Supermix in a final volume of 20 μL. The RT reaction mix was incubated in a thermal cycler (CFX96 Real-Time PCR; Bio-Rad, Hercules, CA) using the following protocol: 5 minutes at 25°C (priming), 20 minutes at 46°C (RT), and 1 minute at 95°C (RT inactivation). Finally, the samples were diluted with Nuclease free water in a final volume of 100 μL and stored at −20°C.

For rt-PCR, we used Go Taq qPCR Master mix (A6102; Promega Corporation, USA) in a CFX96 Touch Real-Time PCR Detection System (Bio-Rad Laboratories). PCR reactions were performed in duplicates for each experimental sample and consisted of 5 μL cDNA, 0.5 μL specific primer forward (fw) 10 nm, 0.5 μL specific primer reverse (rv) 10 nm, 6.25 μL Go Taq qPCR Master mix, and 2.75 μL of Nuclease free water (final volume, 15 μL). Expression of *Mkrn3* was quantified using the following specific primers pairs: r*Mkrn3* fw 5′-AGTTGGACGAAGCAAATCCTC-3′ and r*Mkrn3* rv 5′-AGGTCGTGAGAGTAGCGACA-3′ (accession number XM_218735.10). For data analysis, relative standard curves were constructed from serial dilutions of reference cDNA samples from the whole hypothalamus or MBH, and the input value of the target gene was standardized to the levels of the ribosomal protein S11 (r*Rp-s11*): r*Rp-s11* fw 5′-CATTCAGA CGGAGCGTGCTTAC-3′ and r*Rp-s11* rv 5′-TGCATCTTCATCTTCGTCAC-3′ (accession number NM_031110.1); or hypoxanthine-guanine phosphoribosyltransferase (Hprt): r*Hprt* fw 5′-AGCCGA CCGGTTCTGTCAT-3′ and r*Hprt* rv 5′-GGTCATAACCTGGTTCATCATCAC-3′ (accession number NM_012583.2), used as internal controls. Primer-specific amplification and quantification cycles were run as follows: one cycle of Hot-Start activation at 95°C for 2 minutes, followed by 40 cycles of denaturation at 95°C for 30 seconds; annealing at 60°C for 30 seconds; extension at 72°C for 20 seconds; and a final extension of 72°C for 10 minutes. The dissociation (Melting) curve was used to assess the quality of the PCR. Specificity of PCR products was confirmed by direct sequencing (Central Sequencing Service, Cordoba University). No-template controls were included in all assays.

For analyses of mRNA expression in the mHypoA-55 cell line, total RNA was isolated using PureLink RNA isolation kit (Life Technologies, Burlington, Canada) and RNA concentration was determined using the Nanodrop 2000c spectrophotometer.

For SYBR Green RT-qPCR analyses, contaminating DNA was removed using Turbo DNase (Ambion, Austin, TX) treatment for 15 minutes at room temperature, and 1 μg of RNA was reverse transcribed using the High Capacity cDNA RT Kit (Thermo Fisher Scientific, Waltham, MA) according to the manufacturer’s protocol. Then, 50 ng of cDNA were performed in triplicates and amplified by rt-PCR with Platinum SYBR Green qPCR SuperMix-UDG with ROX (Life Technologies, Burlington, Canada) using an Applied Biosystems 7900 HT Real-Time PCR machine. When measuring Kiss1 mRNA, SensiFAST cDNA Synthesis Kit purchased from Bioline (Toronto, Canada) was used according to the manufacturer’s protocol to make 1 μg of cDNA from total RNA, followed by the use of SensiFAST SYBR Hi-ROX Kit purchased from Bioline containing 1 x SensiFAST SYBR Hi-ROX Mix and 0.3 μM gene-specific primers to amplify 12.5 ng of cDNA template. Amplification of each target gene was performed using the following specific primers pairs: m*Kiss1* fw 5′-TGCTGCTTCTCCTCTGT-3′ and m*Kiss1* rv: 5′-ACCGCGATTCCTTTTCC-3′ (accession number NM_178260.3); m*Mkrn3* fw: 5′-GAGAGGGAA ACGTGCTGTTTA-3′ and m*Mkrn3* rv: 5′-CGGTCATCAGAGAAGGAAGAAC-3′ (accession number NM_011746.3); and m*Histone3a* fw: 5′-CGCTTCCAGAGTGCAGCTATT-3′ and m*Histone3a* rv: 5′-ATCTTCAAAAAGGCCAACCAGAT-3′ (accession number NM_008210.5). Histone 3a served as a housekeeping gene. The PCR conditions used were 1 cycle of Hot-Start activation at 95°C for 2 minutes, followed by 40 cycles of denaturation at 95°C for 30 seconds; annealing at 60°C for 30 seconds; extension at 72°C for 20 seconds, and a final extension of 72 °C for 10 minutes.

For TaqMan RT-qPCR analyses, 1.5 μg of total RNA from the mHypoA-55 cell line was DNAse-treated following the manufacturer’s recommendations (M6101; RQ1 RNase-Free DNase; Promega Corporation, Madison, WI). Each DNAse-treated RNA sample was reverse transcribed using the High Capacity cDNA RT Kit following the manufacturer’s protocol and diluted to a final volume of 200 μL. Then, 2 μL of RT product were amplified using the TaqMan Universal Master Mix II according to the manufacturer’s protocol (Thermo Fisher Scientific, Waltham, MA). rt-PCR was carried out on CFX96 Touch Real-Time PCR Detection System (Bio-Rad Laboratories) and the following TaqMan Gene Expression Assays (Thermo Fisher Scientific, Waltham, MA): *Mkrn3* (Assay ID: Mm00844003_s1), *Kiss1* (Assay ID: Mm03058560_m1), and ribosomal 18s (Assay ID: Hs99999901_s1). Ribosomal 18s served as a housekeeping gene. The PCR conditions used were 1 cycle of polymerase activation at 95°C for 10 minutes, followed by 40 cycles of denaturation at 95°C for 15 seconds, and annealing 60°C for 1 minute.

For analyses of mRNA expression in FACS-sorted Kiss1 neurons, 5 μL of YFP-positive and -negative neurons sorted in extraction buffer (0.1% Triton X-100, Sigma-Aldrich, and 0.4 unit/μL RNaseOUT, Life Technologies) was DNAse-treated using RQ1 RNase-Free DNase (M6101; Promega Corporation, Madison, WI) according to the manufacturer’s recommendations. Each DNAse-treated RNA sample was reverse transcribed using the High Capacity cDNA RT Kit following the manufacturer’s protocol. Then, 2 μL of each RT product were amplified using the TaqMan Universal Master Mix II according to the manufacturer’s protocol (Thermo Fisher Scientific, Waltham, MA). rt-PCR was carried out on CFX96 Touch Real-Time PCR Detection System (Bio-Rad Laboratories) and the following TaqMan Gene Expression Assays (Thermo Fisher Scientific): *Mkrn3* (Assay ID: Mm00844003_s1), *Kiss1* (Assay ID: Mm03058560_m1), *Tac2* (Assay ID: Mm01160362_m1), *Npy* (Assay ID: Mm01410146_m1), and 18s (Assay ID: Hs99999901_s1). Assay 18s served as a housekeeping gene. The PCR conditions used were 1 cycle of Polymerase activation at 95°C for 10 minutes, followed by 40 cycles of denaturation at 95°C for 15 seconds and annealing at 60°C for 1 minute.

For analyses of miRNA expression in hypothalamic tissue and Kiss1 cells, 10 ng of total RNA from hypothalamic rat tissues and the mHypoA-55 cell line or 5 μL of YFP-positive or -negative cell suspension from FACS (in extraction buffer) were reverse transcribed using TaqMan MicroRNA Reverse Transcription Kit (4366596; Applied Biosystems). Each RT reaction consisted of 10 ng total RNA diluted in 5 μL of Nuclease-free water or 5 μL of FACS cell suspensions, 3 μL 5X RT primer, 0.15 μL 100 mM dNTPs (with dTTP), 1 μL MultiScribe reverse transcriptase (50 U/1 μL), 1.50 μL 10X RT buffer, 0.19 μL RNase inhibitor (20 U/μL) and 4.16 μL Nuclease-free water (final volume, 15 μL). This reaction mix was incubated in a thermal cycler (GeneAmp 9700) using the following protocol: 30 minutes at 16°C, 30 minutes at 42°C, and 5 minutes at 85°C. Finally, samples from hypothalamic tissue and mHypoA-55 cells were diluted to a final volume of 215 μL. All samples were stored at −20°C until use for PCR assays.

For quantitative RT-PCR, we used a predesigned assay for miR-30b (Assay Name: hsa-miR-30b; Applied Biosystems). PCR reactions were carried out in a thermal cycler (Roche Light Cycler 480) as follows: 50°C for 2 minutes, 95°C for 10 minutes, followed by 40 cycles of 95°C for 15 seconds and 60°C for 1 minute. For quantitative miRNA determination in hypothalamic rat tissues, *RNU6* served as the internal reference (Assay Name: U6 snRNA; Applied Biosystems).

For immunohistochemistry, infantile female rats (PND10) were anesthetized with ketamine-xylazine and perfused intracardially with saline (0.9% NaCl) followed by 4% PFA in PBS (pH 7.4). Fixed brains were immersed in 30% sucrose and 0.01% sodium azide in PBS at 4°C for 2–4 days. Next, 3 sets of coronal, 40-μm-thick sections were cut in a freezing microtome Leica CM1850 UV and stored at −20°C in cryoprotectant. For immunodetection of the target protein, Mkrn3, one set of sections encompassing the whole hypothalamus was used from each animal, and standard procedures for single-label immunohistochemistry were performed (*see* below).

For analysis of the neuroanatomical distribution of Mkrn3 protein expression in the hypothalamus, one set of free-floating brain sections from infantile female rats (PND10) were (i) washed in Tris-buffered saline TBS (pH 7.6) (3 × 10 minutes) at room temperature with gentle agitation; (ii) incubated with blocking solution containing the primary antibody anti-MKRN3 (1:1,000; Rabbit Anti-MKRN3/RNF63 Polyclonal Antibody; TA349159, Origene) at 4°C for 72 hours; (iii) washed in TBS (pH 7.6) (3 × 10 minutes) at room temperature; (iv) incubated with a secondary biotinylated donkey anti-rabbit antibody (1:500; JAC-711-066-152, Jackson Immunoresearch) for 90 minutes at room temperature; (v) washed (3 × 10 minutes) in TBS (pH 7.6); (vi) incubated with A/B Vectastain Elite solution (VECTASTAIN Elite ABC Kit reagents; Vector Laboratories, Burlingame, CA) at room temperature for 90 minutes; (vii) washed (3 × 5 minutes) in TBS (pH 7.6) and acetate buffer 0.1 M (3 × 5 minutes); and (viii) incubated with glucose oxidase and diaminobenzidine-nickel (DAB/Ni) for 20 minutes at room temperature. After several washes in acetate buffer 0.1 M and TSB (pH 7.6), brain sections were air-dried and dehydrated in ascending concentrations of alcohol (50, 70, 95, 100, 100) and xylene, and coverslip using Eukitt mounting medium (MICROPTIC S.L., Barcelona, Spain). Immunoreactivity was visualized in a microscope Leica DM2500 using a 10× lens.

For WB, total protein was extracted from the whole hypothalamus as previously described. Briefly, total protein lysates (40 μg) were subjected to SDS-PAGE on 7% polyacrylamide gels, electro-transferred on polyvinylidene difluoride (PVDF) membranes (Millipore), and probed overnight at 4°C in the presence of the primary antibody anti-MKRN3 (1:500 dilution, TA349159, Origene). For protein detection, we used horseradish peroxidase–conjugated secondary antibodies and chemiluminescence (Abcam). Samples (4–6 per group) were assayed; protein levels were normalized to β-actin (1:5,000 dilution, A5060, Sigma Aldrich). Densitometic analysis of protein bands was conducted using the open-source image processing software, ImageJ (https://imagej.net/ImageJ).

For radioimmunoassay (RIA), serum LH and FSH levels were measured using RIA kits supplied by the National Institutes of Health (Dr. A. F. Parlow, National Hormone and Peptide Program, Torrance, CA). Hormonal determinations were performed in duplicates. Rat LH-I-10 and FSH-I-9 were labeled with ^125^I by the chloramine-T method, and hormone concentrations were expressed using reference preparations LH-RP-3 and FSH-RP-2 as standards. Intra- and inter-assay coefficients of variation were 8% and 10% for LH and 6% and 9% for FSH, respectively. The sensitivity of the assay was 5 pg/tube for LH and 20 pg/tube for FSH. Accuracy of hormone determinations was confirmed by assessment of rat serum samples of known concentrations (used as internal quality controls).

#### Bioinformatic analyses

In order to identify potential regulatory miRNAs of the *Mkrn3* transcript, we used four miRNA target prediction tools based on different methods: (i) *miRanda*, type of method: complementary, resource: http://www.mirbase.org/ [43]; (ii) *Target-Scan*, type of method: seed complementary, resource: http://www.targetscan.org [44]; *PicTar*, type of method: thermodynamics, resource: http://pictar.mdc-berlin.de/ [45]; and *MiRtarget2*, type of method: support vector machine, resource: http://mirdb.org [46].

### Statistical analyses

Statistical analyses were performed using Prism software (Graphpad Prism 6.0 for Macintosh, GraphPad Software, La Jolla, CA, www.graphpad.com). The differences between several groups were analyzed by one-way ANOVA followed by the Tukey multiple comparison test for unequal replications. When comparing the influence of two different independent variables, experimental groups were subjected to a two-way ANOVA test followed by Sidak's multiple comparisons test. The Student *t* test was used to compare two groups. As a general procedure, the group sizes were selected based on previous experience with studies addressing molecular and neuroendocrine regulation of puberty, assisted by power analyses performed using values of standard deviation that we usually obtain when measuring parameters analogous to those examined in this study. Based on those calculations, a minimal group size of *n* = 8 animals per group was established as general rule, as analyses using these sample size should provide at least 80% power to detect effect sizes using the tests indicated above. For physiological experiments, group sizes usually exceeded this limit. However, based on standard procedures, while phenotypic and hormonal analyses were applied to all individuals, more complex molecular and histological analyses in these experiments were implemented in a representative subset of randomly assigned samples from each group. Thus, hormonal determinations were performed in duplicate with a minimal total number of 8–12 determinations per group, RNA analyses were performed in duplicate from at least 5 independent samples per group, and WBs were conducted with at least 4–6 samples per group. All data are expressed as the mean ± SEM for each group; a *P* value of <0.05 was considered statistically significant. As a general procedure, the investigators directly performing experimentation were not blinded to the group allocation, but primary data analyses by senior authors were conducted independently to avoid any potential bias.

## Supporting information

S1 Fig Expression profiles of Mkrn3 mRNA and miR-30b in whole hypothalamic fragments of female (**A**) and male (**B**) rats during postnatal maturation (*n* = 5–9/group). Representative photomicrographs of Mkrn3 immunoreactivity (ir) in the hypothalamus of infantile female rats (PND10) are shown (**C**). Specific neuroanatomical distribution of Mkrn3-ir in the hypothalamic ARC is presented at higher magnification (**C**; lower panel). Data are presented as mean ± SEM. Different superscript letters above bars indicate statistical differences; one-way ANOVA followed by post hoc Tukey test. For underlying data, see S1 Data file. ARC, arcuate nucleus; Mkrn3, makorin RING-finger protein-3; PND, postnatal day.
(TIF)

S2 Fig Phenotypic and hormonal parameters of normal pubertal maturation in female (upper panels) and male (lower panels) rats. In females, the cumulative percentage and mean age of VO, as well as the gonadotropin levels (LH and FSH) at the day of VO are shown in (**A**). In addition, the cumulative percentage and mean age of first estrus (FE), as well as the gonadotropin levels and reproductive organ weights (ovary and uterus) at the day of FE are presented in (**B**) (*n* ≥ 10/group). In males, the cumulative percentage and mean age of PS, as well as the gonadotropin levels and reproductive organ weights (testis and prostate) at the day of PS are presented in (**C**) (*n* ≥ 12/group). For underlying data, see S1 Data file. FSH, follicle-stimulating hormone; LH, luteinizing hormone; PS, preputial separation; VO, vaginal opening.
(TIF)

S3 Fig Schematic representation of the generation of the clonal, immortalized hypothalamic cell line, mHypoA55, from the ARC of adult female mice (**A**). The threshold cycle (Ct) number in rt-PCR assays for Histone-3 (used as housekeeping), Mkrn3, and Kiss1 transcripts, obtained using SYBR Green detection, in mHypoA55 cells (<15 passages) are shown (**B**). In addition, rt-PCR assays using specific TaqMan probes for the housekeeping 18s, Mkrn3, and Kiss1 transcripts, as well as miR-30b, were performed in mHypoA55 cells (>15 passages); amplification curves for each target are shown in (**C**), where the automatically set threshold limit of detection is represented by a continuous line. For underlying data, see S1 Data file. ARC, arcuate nucleus; Mkrn3, makorin RING-finger protein-3; rt, real-time.
(TIF)

S4 Fig Schematic representation of the procedure of isolation of ARC Kiss1 neurons by FACS from MBH (including the ARC), microdissected from the Kiss1-Cre/YFP mouse line at PND29 (**A**). Forward versus side scatter (FSC versus SSC) gating was used to identify cells of interest based on size (FSC) and structure (SSC) (**B**). A forward scatter height (FSC-H) versus forward scatter area (FSC-A) density plot was used to exclude the aggregated cells (**C**). There was lack of detection of YFP-positive cells in cortex, used as negative control (**D**), while a fraction of YFP-positive cells appeared in the ARC of the same mouse (**E**). rt-PCR analysis of the expression of 18s (used as housekeeping), Mkrn3, Kiss1, Tac2, and Npy transcripts, as well as of miR-30b, was conducted in ARC YFP-positive and YFP-negative FAC-sorted cells using specific TaqMan probes; amplification curves for each target are shown in (**F**, YFP-positive) and (**G**, YFP-negative), where the automatically set threshold limit of detection is represented by a continuous line. For underlying data, see S1 Data file. ARC, arcuate nucleus; FACS, fluorescence-activated cell sorting; MBH, medial-basal hypothalamus; Mkrn3, makorin RING-finger protein-3; Npy, Neuropeptide Y; PND, postnatal day; rt, real-time; YFP, yellow fluorescent protein.
(TIF)

S5 Fig Phenotypic pubertal markers in a rat model of perturbed puberty due to neonatal estrogenization. Cumulative percentages of VO (**A**) and first estrus (**B**) of female rats neonatally injected (on PND1) with EB are presented. Animals injected with olive oil (VEH) served as controls (*n* = 10/group). For underlying data, see S1 Data file. EB, estradiol benzoate; PND, postnatal day; VEH, vehicle; VO, vaginal opening.
(TIFF)

S6 Fig Expression profiles of Mkrn3 mRNA and miR-30b in the hypothalamus of female rats subjected to early postnatal underfeeding (LL). Expression analyses were conducted at PND5, 15, and 35. Animals bred in NLs served as controls (*n* = 6–8/group). Data are presented as mean ± SEM. *P ≤ 0.05 versus corresponding PND5 NL; two-way ANOVA followed by post hoc Sidak’s test. For underlying data, see S1 Data file. LL, large litter; Mkrn3, makorin RING-finger protein-3; NL, normal litter; PND, postnatal day.
(TIF)

S7 Fig Strategy used to selectively prevent the binding of miR-30 to its seed regions at the 3′ UTR of *Mkrn3*, using TSBs in vivo. A schematic of the experimental protocol of repeated central (icv) administration of a mix of TSB-miR-30 during the juvenile or the prepubertal period is shown in (**A**). In addition, a diagram showing the three different TSB-miR-30 tailored to block each of the seed regions of miR-30 in the 3′ UTR of Mkrn3 is presented in (**B**). icv, intracerebroventricular; *Mkrn3*, makorin RING-finger protein-3; TSB, Target Site Blocker.
(TIFF)

S8 Fig Impact of prepubertal icv administration of TSB-miR-30 on the timing of puberty onset in female rats. The effects of prepubertal icv treatment with TSB-miR-30 on BW and food intake (**A**), as well as relevant reproductive parameters, including the cumulative percentage of VO and first estrus (**B**), and ovarian weight (OW) and uterus weight (UW) (**C**) are presented (*n* = 10–12/group). In addition, densitometric quantification and a representative WB autoradiographic image of Mkrn3 protein from hypothalamic samples of pubertal female rats subjected to prepubertal icv administration of TSB-miR-30 are shown (**D**; *n* = 5/group). Loading control (β-Actin) is also presented. Females icv injected with vehicle (VEH) served as controls. For underlying data, see S1 Data file. icv, intracerebroventricular; Mkrn3, makorin RING-finger protein-3; TSB, Target Site Blocker; VO, vaginal opening; WB, western blot.
(TIF)

S1 Data (XLSX)

S1 Raw Images Western blot. (PDF)

## Abbreviations

ARC: arcuate nucleus
BW: body weight
CL: corpora lutea
CMV: cytomegalovirus
DAB/Ni: diaminobenzidine-nickel
DREAM: downstream responsive element antagonist modulator
EB: estradiol benzoate
eGFP: enhanced green fluorescent protein
EYFP: enhanced yellow-fluorescent protein
FACS: fluorescence-activated cell sorting
FBS: fetal bovine serum
FSC-A: forward scatter
FSH: follicle-stimulating hormone
GnRH: gonadotropin-releasing hormone
Hprt: hypoxanthine-guanine phosphoribosyltransferase
icv: intracerebroventricular
LH: luteinizing hormone
LL: large litter
MBH: medial-basal hypothalamus
miRNA: microRNA
Mkrn3: makorin RING-finger protein-3
NKB: neurokinin B
NL: normal litter
Nptx1: neural pentraxin-1 precursor
Npy: neuropeptide Y
PEI: polyethylenimine
PND: postnatal day
PS: preputial separation
PVDF: polyvinylidene difluoride
qPCR: quantitative polymerase chain reaction
RIA: radioimmunoassay
r*Rp-s11*: ribosomal protein S11
rt: real-time
RT: reverse transcription
sc: subcutaneously
SSC-A: side scatter
SV40: simian virus 40
TSB: Target Site Blocker
VO: vaginal opening
WB: western blot
YFP: yellow fluorescent protein
