# Supplementary figures and images for: Hypothalamic miR-30 regulates puberty onset via repression of the puberty-suppressing factor, Mkrn3
Source: PLoS Biol. 2019 Nov 7;17(11):e3000532. doi: 10.1371/journal.pbio.3000532 (PMC6863565; doi:10.1371/journal.pbio.3000532)

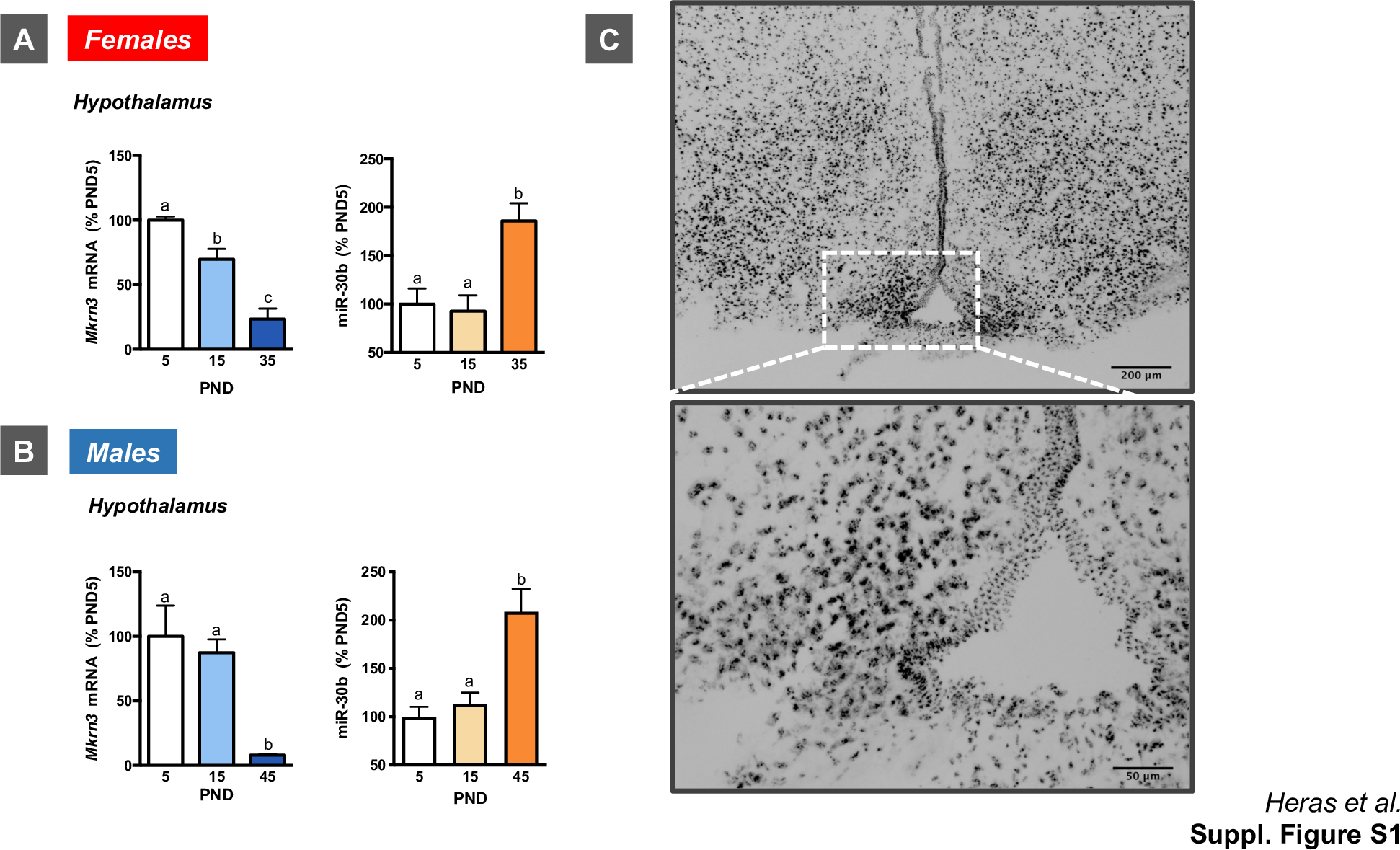

Supplement: S1 Fig — Expression profiles of Mkrn3 mRNA and miR-30b in whole hypothalamic fragments of female (A) and male (B) rats during postnatal maturation (n = 5–9/group). Representative photomicrographs of Mkrn3 immunoreactivity (ir) in the hypothalamus of infantile female rats (PND10) are shown (C). Specific neuroanatomical distribution of Mkrn3-ir in the hypothalamic ARC is presented at higher magnification (C; lower panel). Data are presented as mean ± SEM. Different superscript letters above bars indicate statistical differences; one-way ANOVA followed by post hoc Tukey test. For underlying data, see S1 Data file. ARC, arcuate nucleus; Mkrn3, makorin RING-finger protein-3; PND, postnatal day. (TIF) [file pbio.3000532.s001.tif]

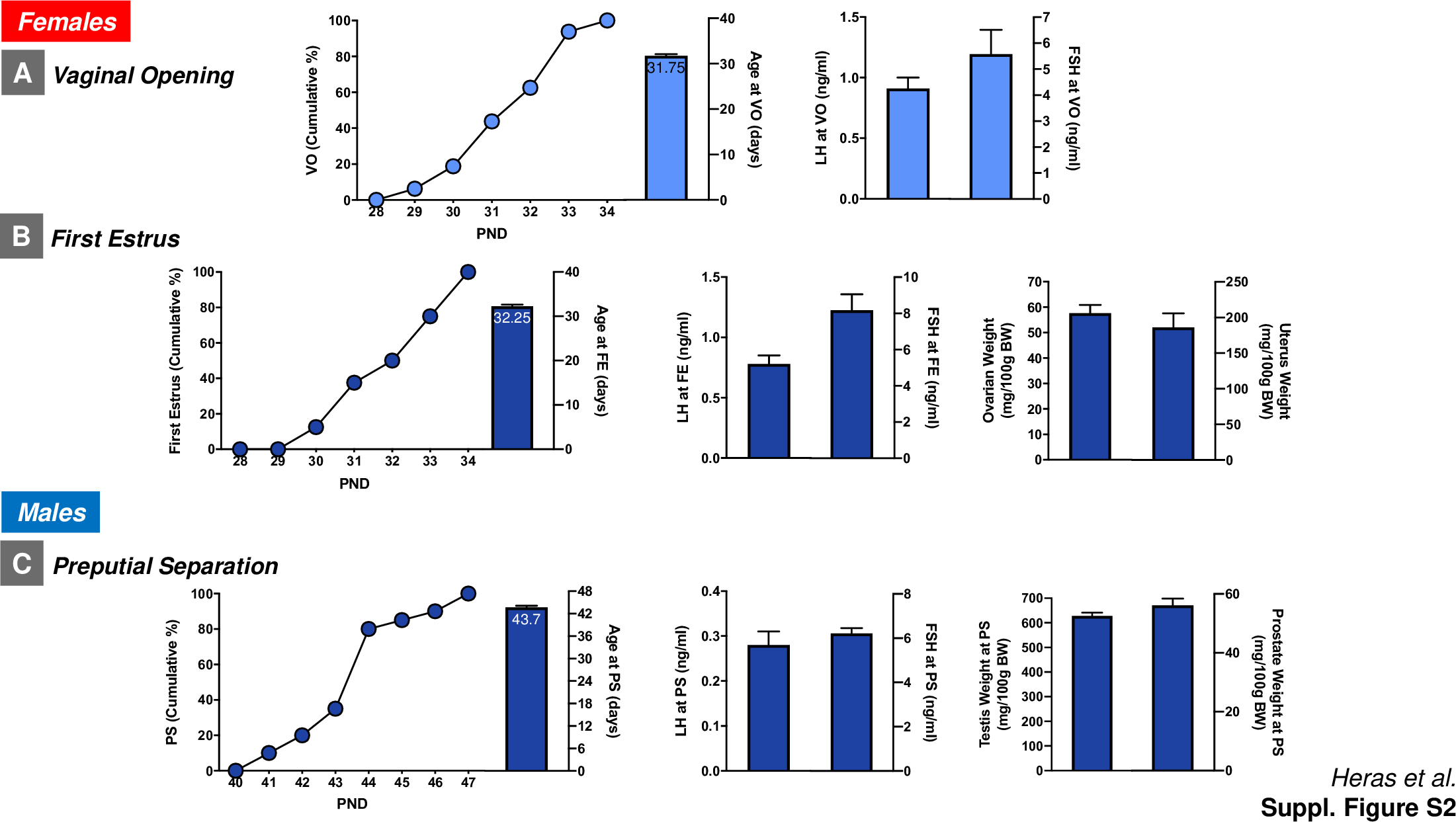

Supplement: S2 Fig — Phenotypic and hormonal parameters of normal pubertal maturation in female (upper panels) and male (lower panels) rats. In females, the cumulative percentage and mean age of VO, as well as the gonadotropin levels (LH and FSH) at the day of VO are shown in (A). In addition, the cumulative percentage and mean age of first estrus (FE), as well as the gonadotropin levels and reproductive organ weights (ovary and uterus) at the day of FE are presented in (B) (n ≥ 10/group). In males, the cumulative percentage and mean age of PS, as well as the gonadotropin levels and reproductive organ weights (testis and prostate) at the day of PS are presented in (C) (n ≥ 12/group). For underlying data, see S1 Data file. FSH, follicle-stimulating hormone; LH, luteinizing hormone; PS, preputial separation; VO, vaginal opening. (TIF) [file pbio.3000532.s002.tif]

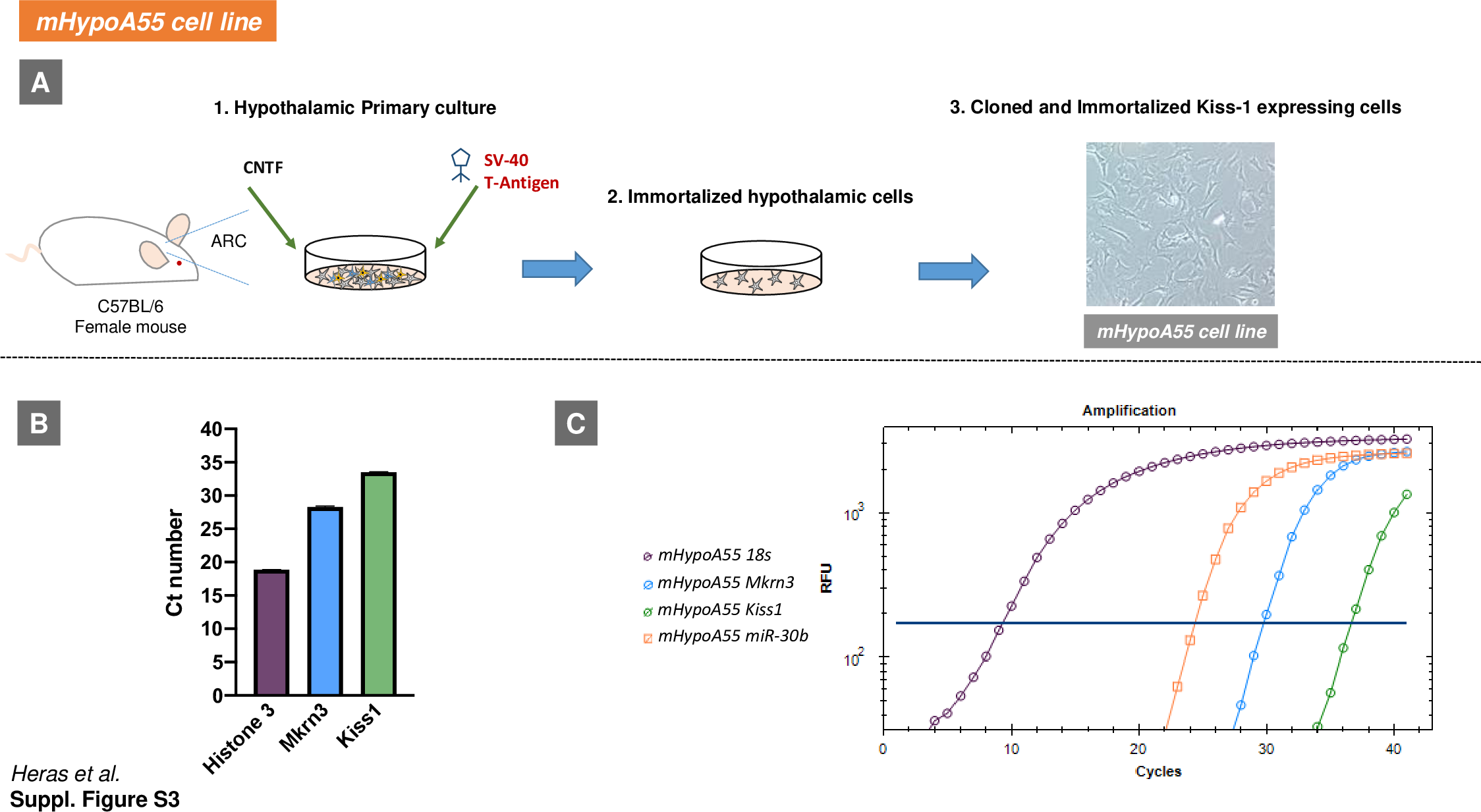

Supplement: S3 Fig — Schematic representation of the generation of the clonal, immortalized hypothalamic cell line, mHypoA55, from the ARC of adult female mice (A). The threshold cycle (Ct) number in rt-PCR assays for Histone-3 (used as housekeeping), Mkrn3, and Kiss1 transcripts, obtained using SYBR Green detection, in mHypoA55 cells (<15 passages) are shown (B). In addition, rt-PCR assays using specific TaqMan probes for the housekeeping 18s, Mkrn3, and Kiss1 transcripts, as well as miR-30b, were performed in mHypoA55 cells (>15 passages); amplification curves for each target are shown in (C), where the automatically set threshold limit of detection is represented by a continuous line. For underlying data, see S1 Data file. ARC, arcuate nucleus; Mkrn3, makorin RING-finger protein-3; rt, real-time. (TIF) [file pbio.3000532.s003.tif]

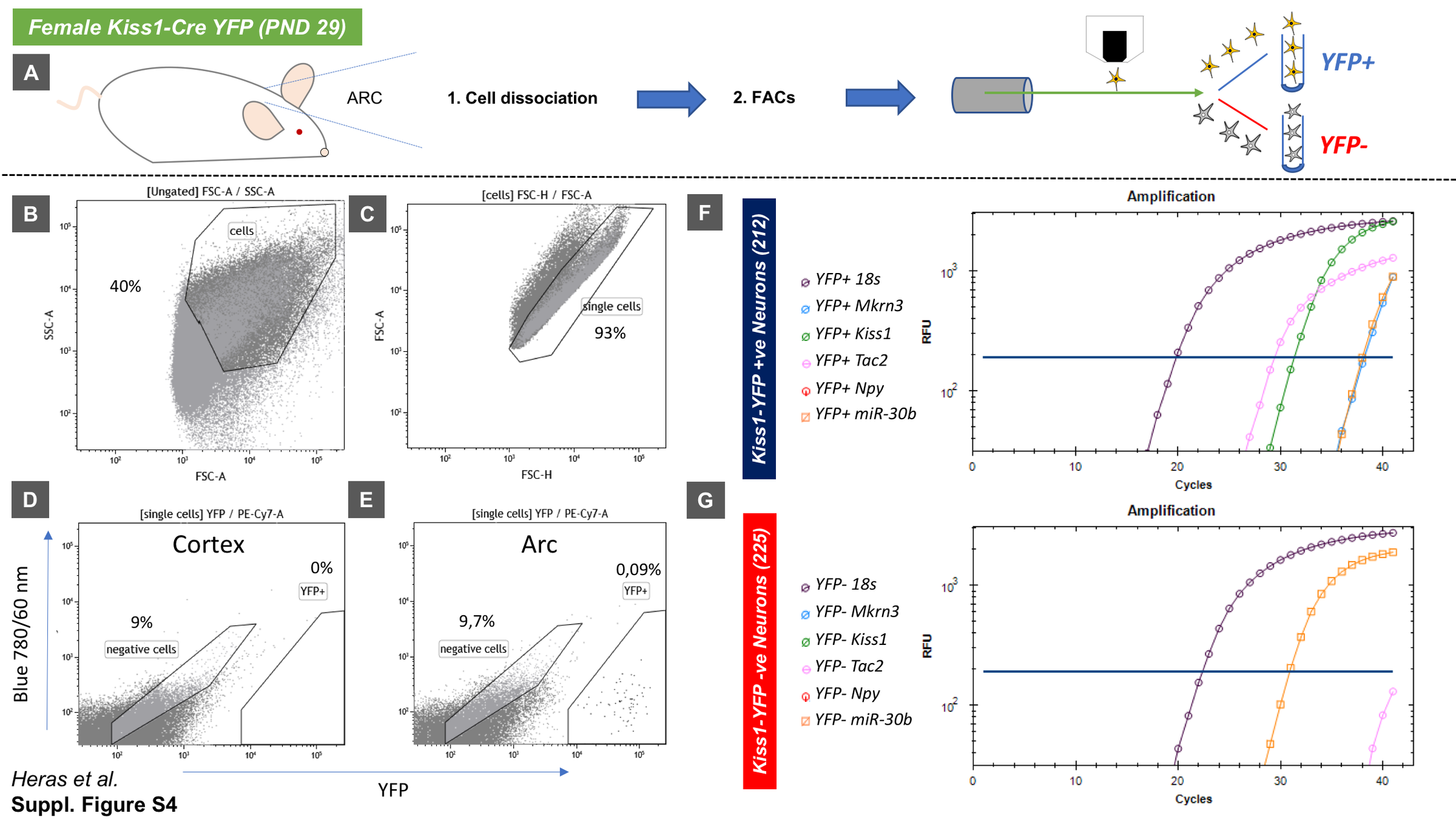

Supplement: S4 Fig — Schematic representation of the procedure of isolation of ARC Kiss1 neurons by FACS from MBH (including the ARC), microdissected from the Kiss1-Cre/YFP mouse line at PND29 (A). Forward versus side scatter (FSC versus SSC) gating was used to identify cells of interest based on size (FSC) and structure (SSC) (B). A forward scatter height (FSC-H) versus forward scatter area (FSC-A) density plot was used to exclude the aggregated cells (C). There was lack of detection of YFP-positive cells in cortex, used as negative control (D), while a fraction of YFP-positive cells appeared in the ARC of the same mouse (E). rt-PCR analysis of the expression of 18s (used as housekeeping), Mkrn3, Kiss1, Tac2, and Npy transcripts, as well as of miR-30b, was conducted in ARC YFP-positive and YFP-negative FAC-sorted cells using specific TaqMan probes; amplification curves for each target are shown in (F, YFP-positive) and (G, YFP-negative), where the automatically set threshold limit of detection is represented by a continuous line. For underlying data, see S1 Data file. ARC, arcuate nucleus; FACS, fluorescence-activated cell sorting; MBH, medial-basal hypothalamus; Mkrn3, makorin RING-finger protein-3; Npy, Neuropeptide Y; PND, postnatal day; rt, real-time; YFP, yellow fluorescent protein. (TIF) [file pbio.3000532.s004.tif]

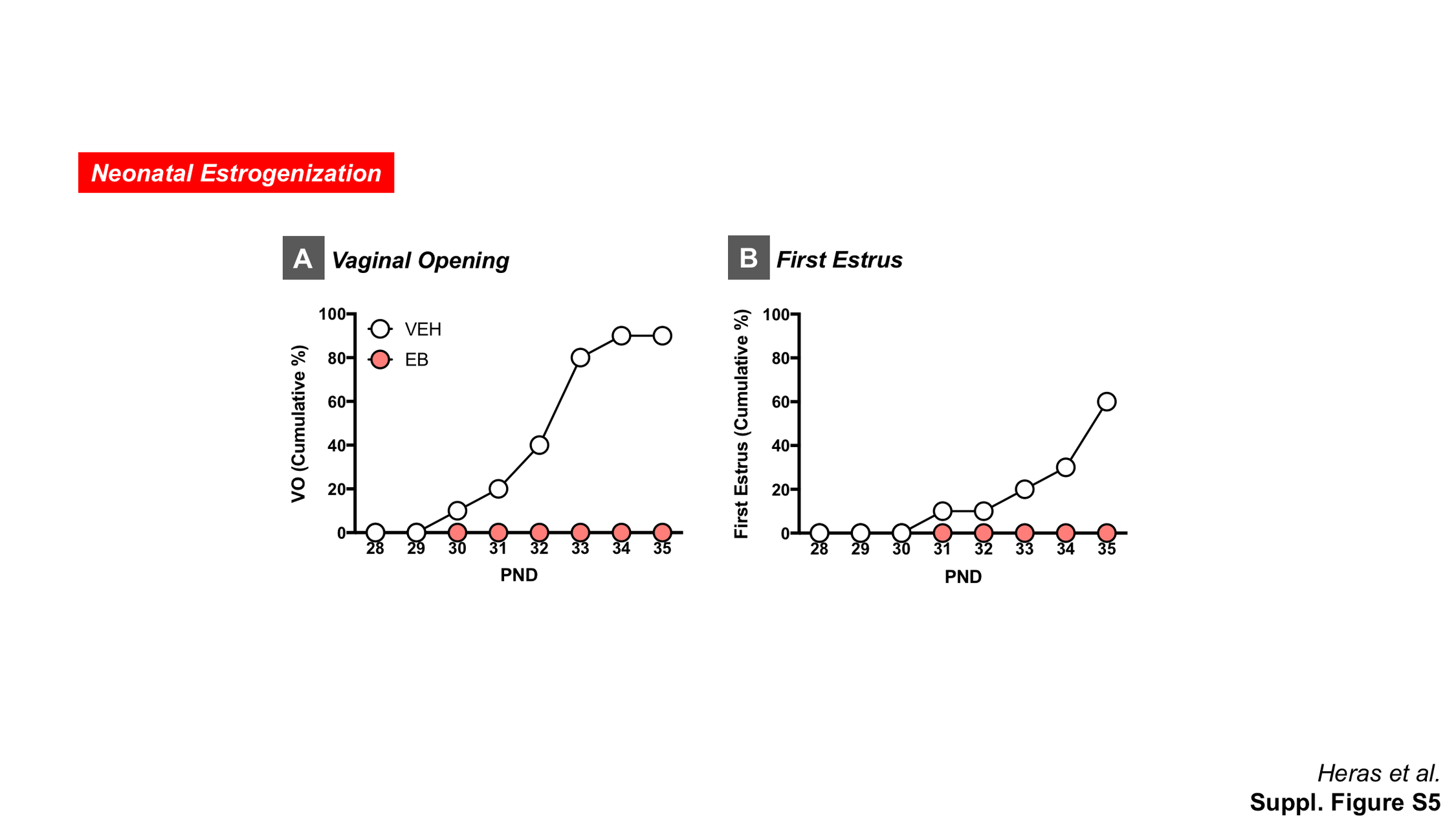

Supplement: S5 Fig — Cumulative percentages of VO (A) and first estrus (B) of female rats neonatally injected (on PND1) with EB are presented. Animals injected with olive oil (VEH) served as controls (n = 10/group). For underlying data, see S1 Data file. EB, estradiol benzoate; PND, postnatal day; VEH, vehicle; VO, vaginal opening. (TIFF) [file pbio.3000532.s005.tiff]

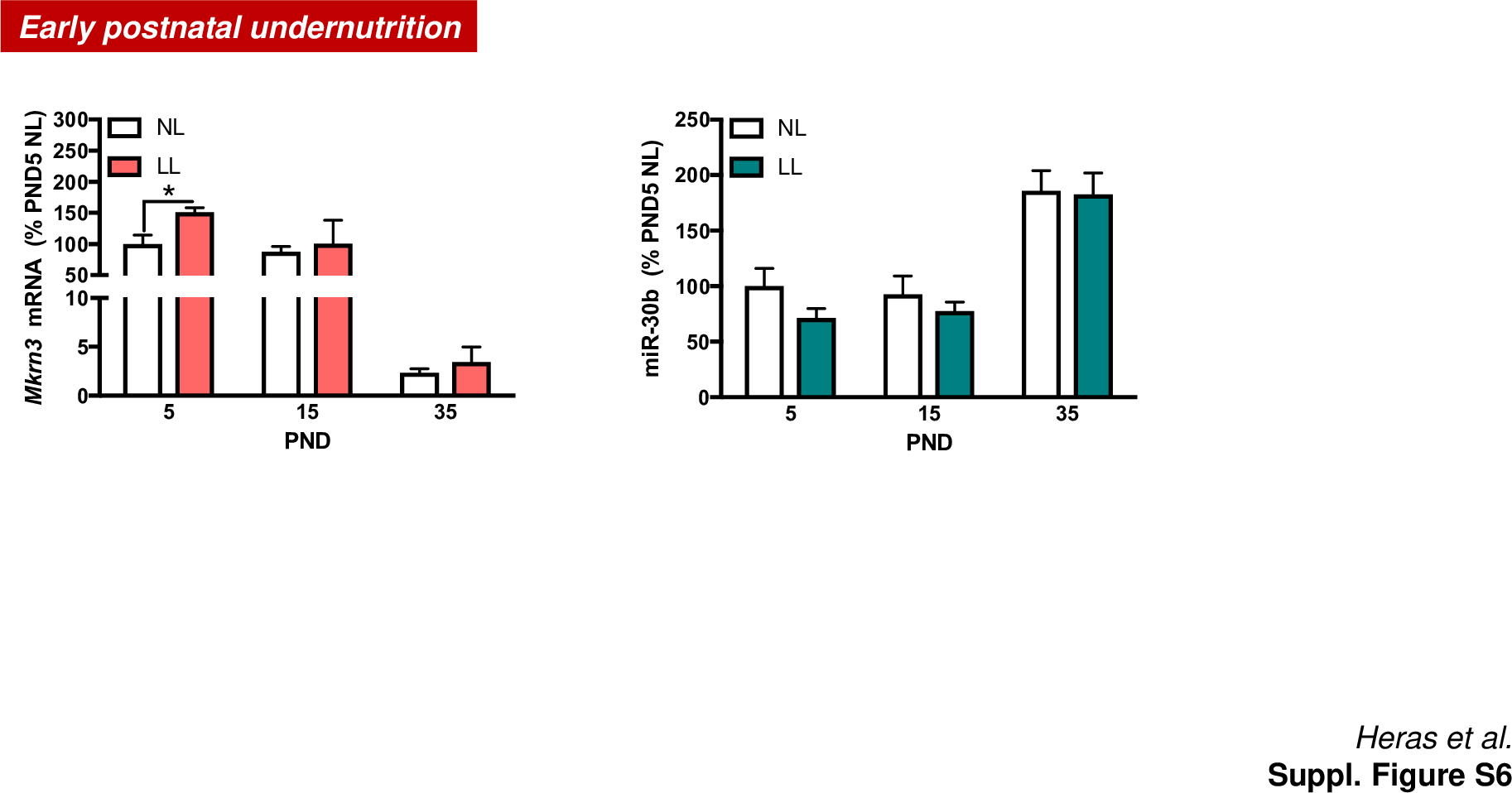

Supplement: S6 Fig — Expression analyses were conducted at PND5, 15, and 35. Animals bred in NLs served as controls (n = 6–8/group). Data are presented as mean ± SEM. *P ≤ 0.05 versus corresponding PND5 NL; two-way ANOVA followed by post hoc Sidak’s test. For underlying data, see S1 Data file. LL, large litter; Mkrn3, makorin RING-finger protein-3; NL, normal litter; PND, postnatal day. (TIF) [file pbio.3000532.s006.tif]

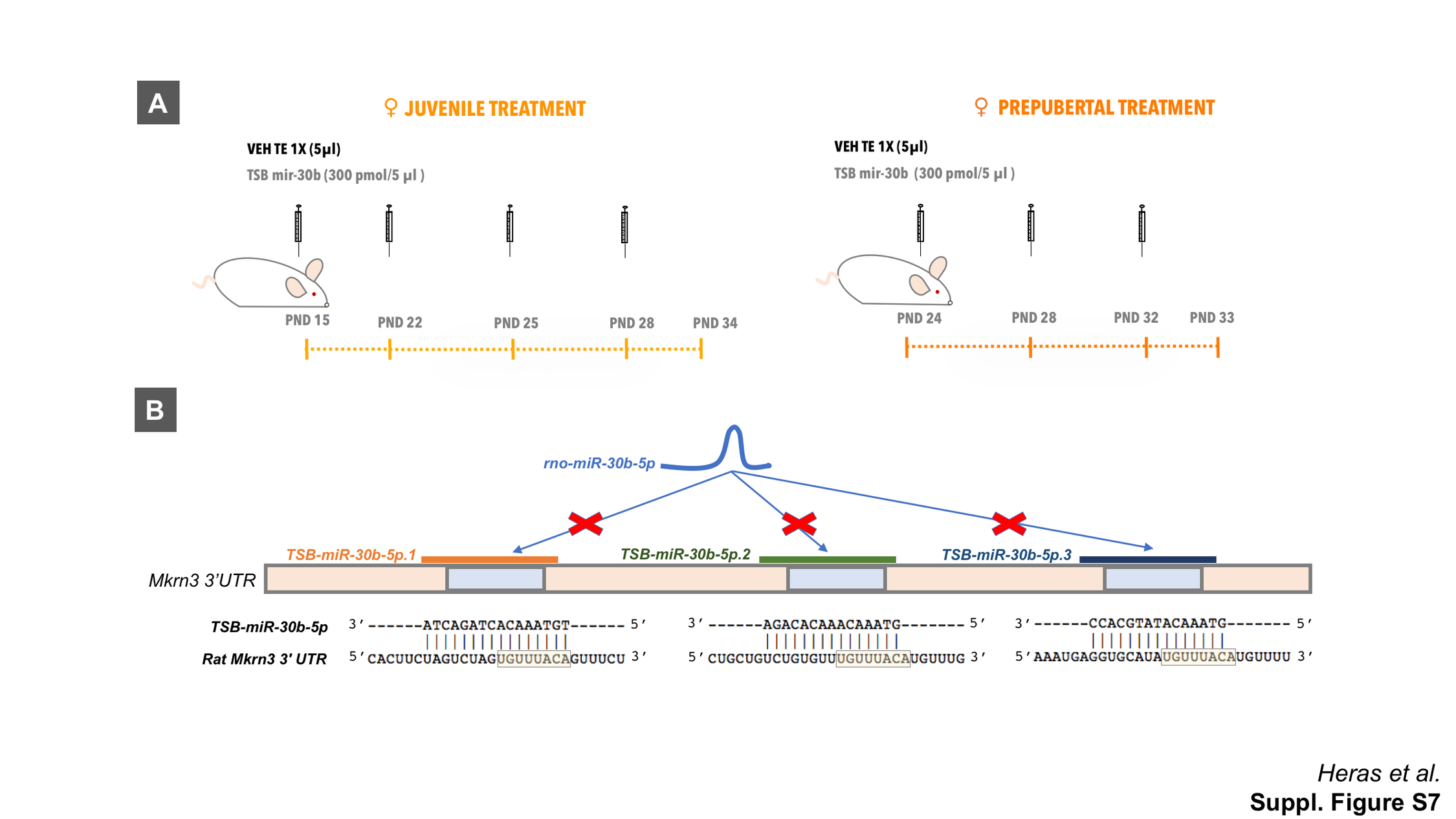

Supplement: S7 Fig — A schematic of the experimental protocol of repeated central (icv) administration of a mix of TSB-miR-30 during the juvenile or the prepubertal period is shown in (A). In addition, a diagram showing the three different TSB-miR-30 tailored to block each of the seed regions of miR-30 in the 3′ UTR of Mkrn3 is presented in (B). icv, intracerebroventricular; Mkrn3, makorin RING-finger protein-3; TSB, Target Site Blocker. (TIFF) [file pbio.3000532.s007.tiff]

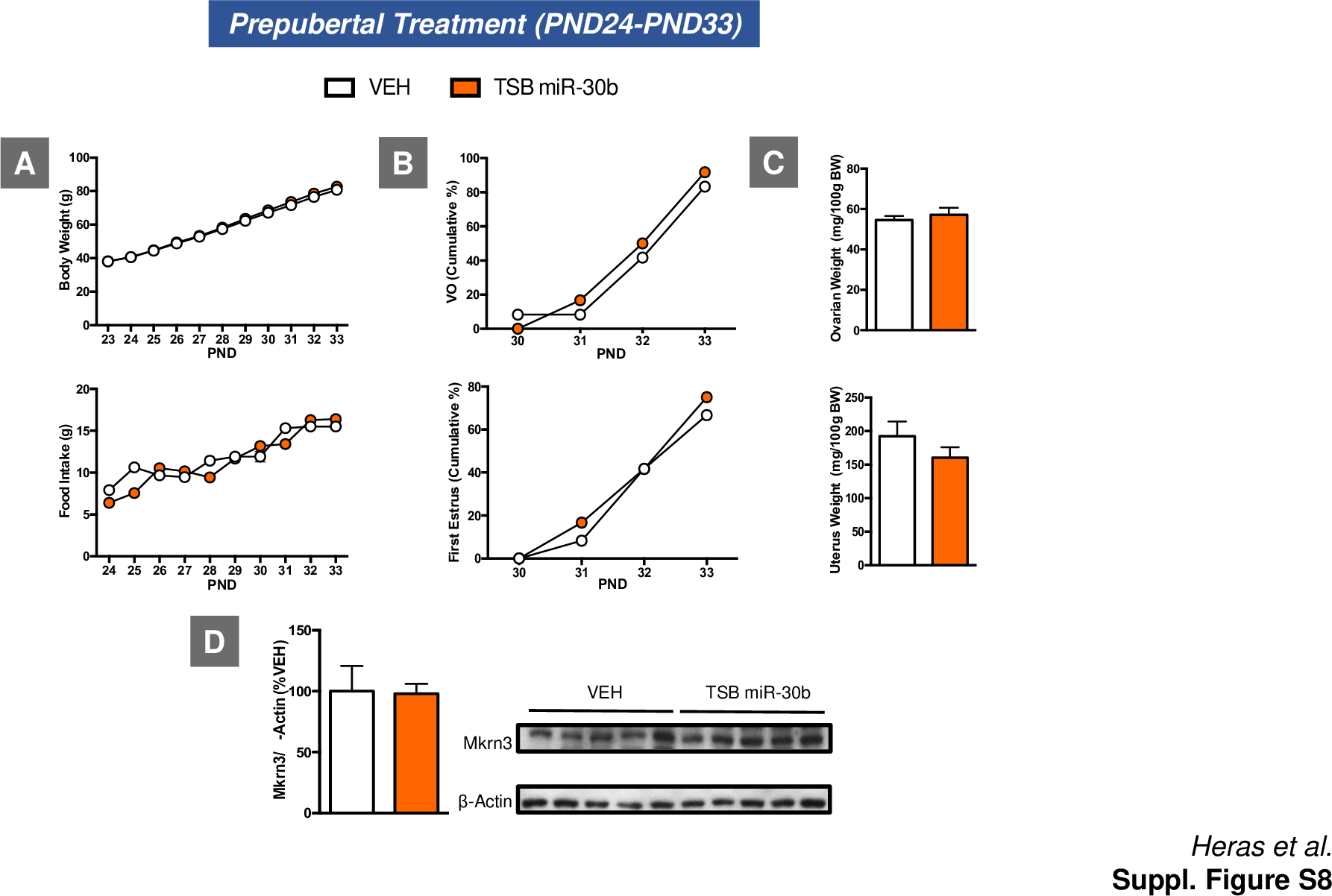

Supplement: S8 Fig — The effects of prepubertal icv treatment with TSB-miR-30 on BW and food intake (A), as well as relevant reproductive parameters, including the cumulative percentage of VO and first estrus (B), and ovarian weight (OW) and uterus weight (UW) (C) are presented (n = 10–12/group). In addition, densitometric quantification and a representative WB autoradiographic image of Mkrn3 protein from hypothalamic samples of pubertal female rats subjected to prepubertal icv administration of TSB-miR-30 are shown (D; n = 5/group). Loading control (β-Actin) is also presented. Females icv injected with vehicle (VEH) served as controls. For underlying data, see S1 Data file. icv, intracerebroventricular; Mkrn3, makorin RING-finger protein-3; TSB, Target Site Blocker; VO, vaginal opening; WB, western blot. (TIF) [file pbio.3000532.s008.tif]
