# Supplementary material for: Hypothalamic miR-30 regulates puberty onset via repression of the puberty-suppressing factor, Mkrn3
Source: PLoS Biol. 2019 Nov 7;17(11):e3000532. doi: 10.1371/journal.pbio.3000532 (PMC6863565; doi:10.1371/journal.pbio.3000532)
Supplement: S1 Raw Images — (PDF) [file pbio.3000532.s010.pdf]

Gel 1A

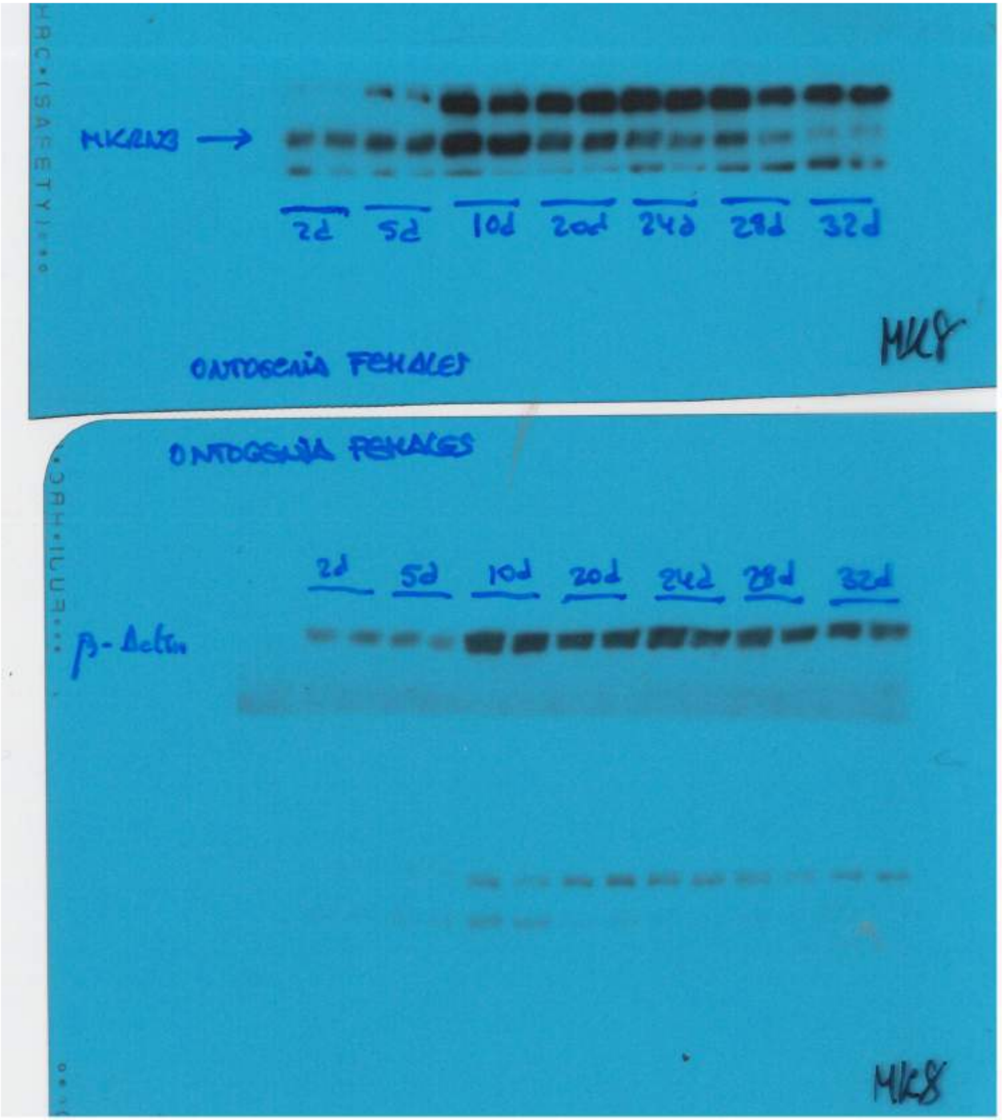

Gel 1B

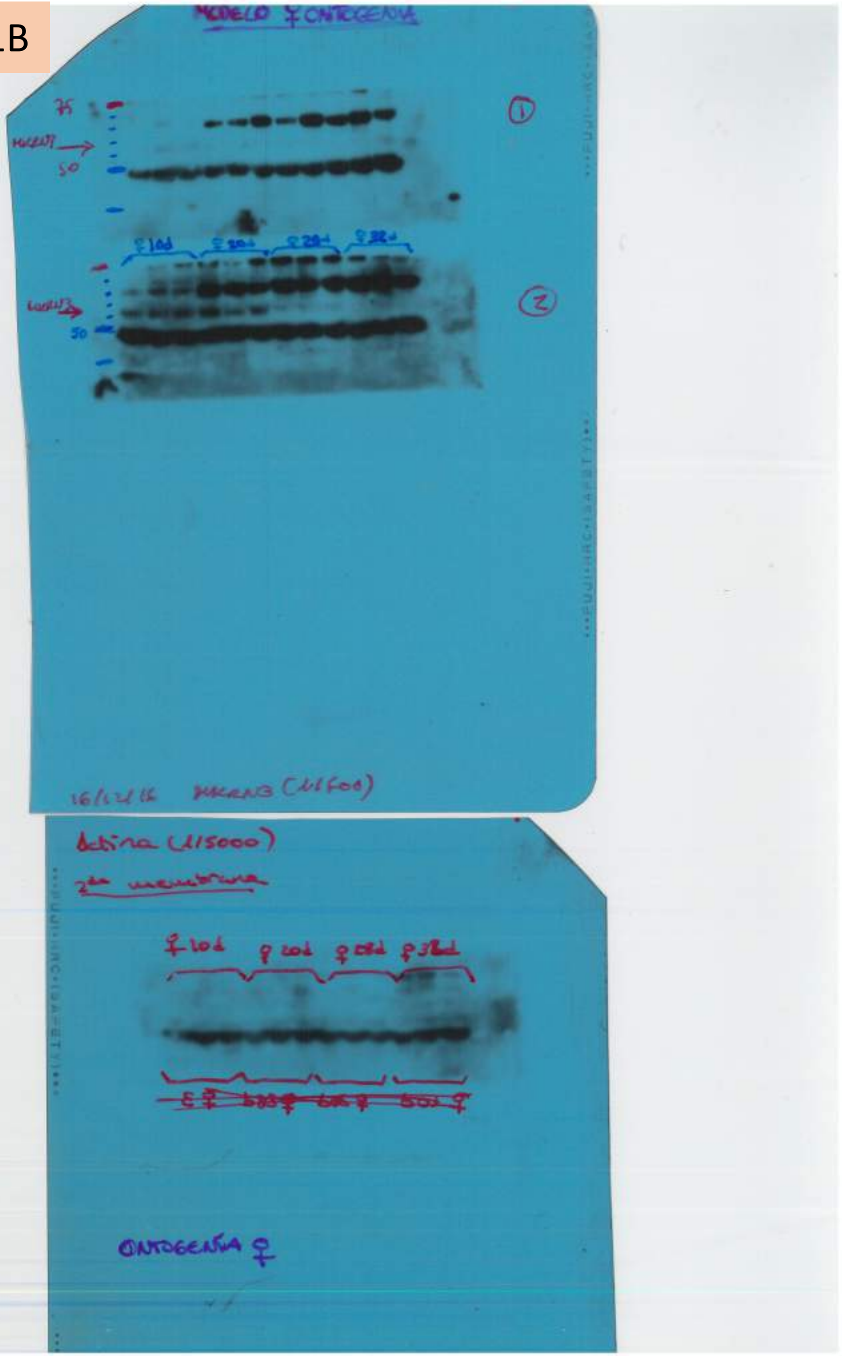

Uncropped WB Figure 2E

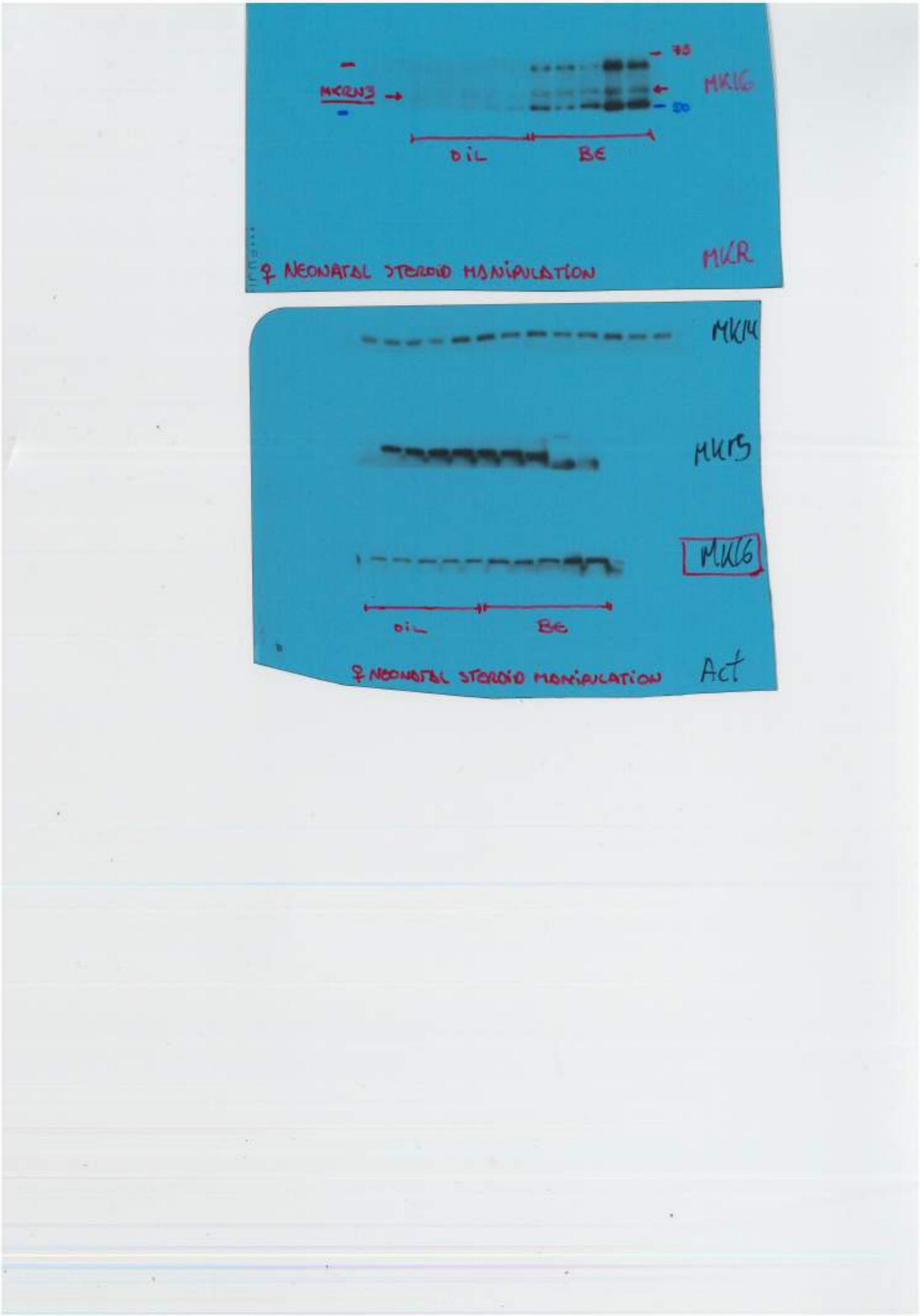

Uncropped WB Figure 3C

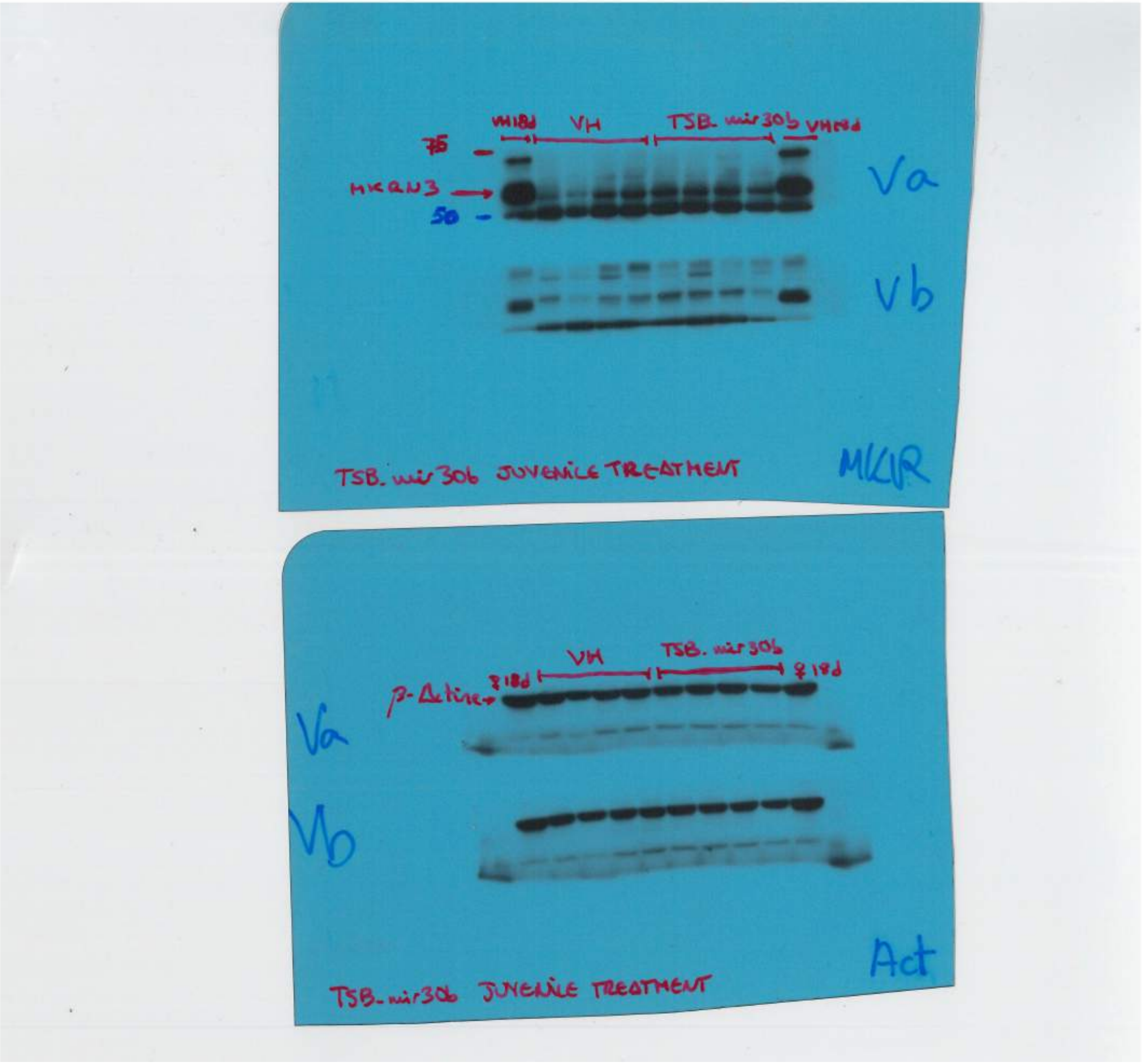

Uncropped WB Figure 5F

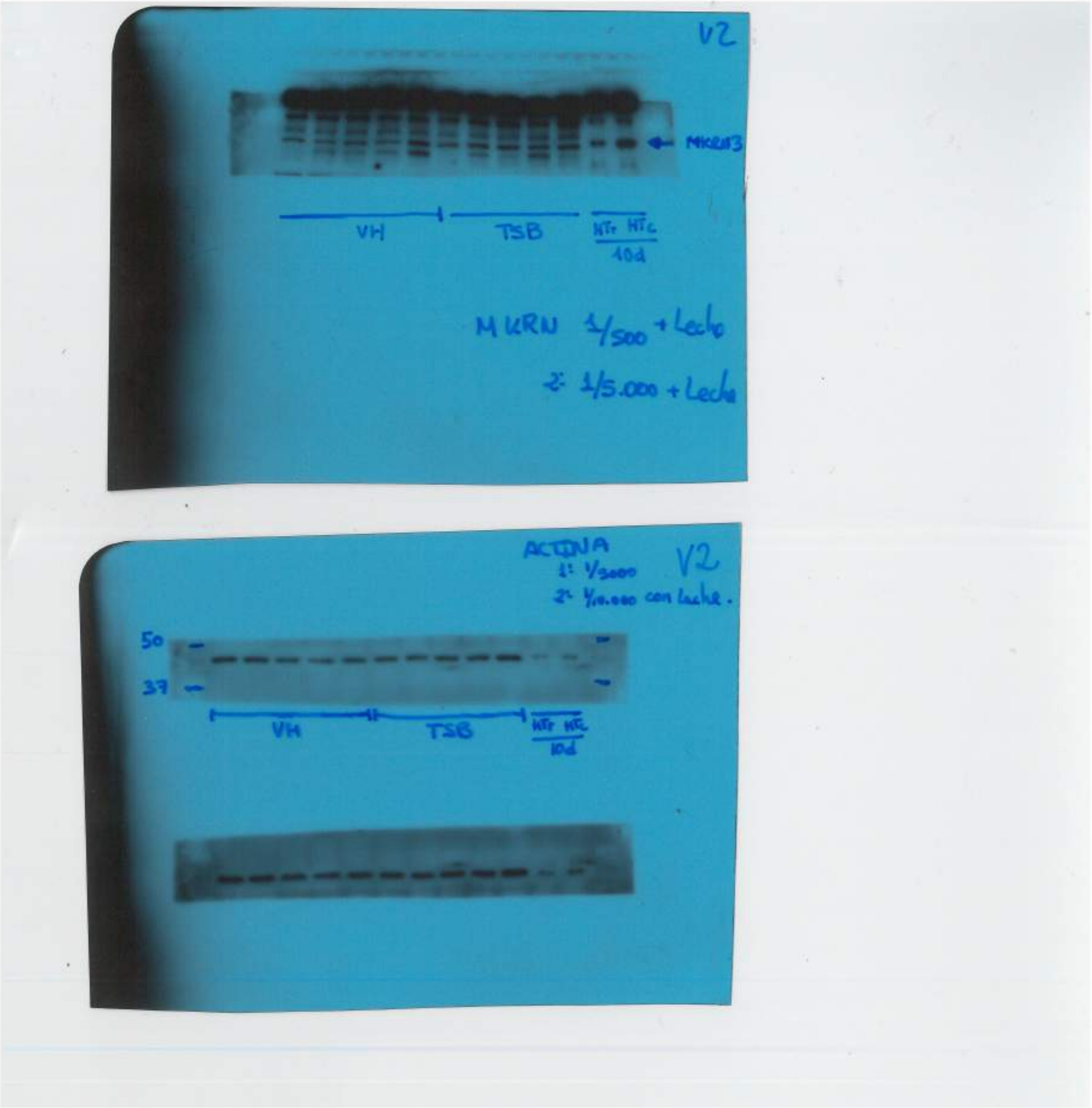

Uncropped WB Suppl. Figure 8D
